# Identification and Genetic Diversity Analysis of the Pathogen of Anthracnose of Pepper in Guizhou

**DOI:** 10.3390/plants13050728

**Published:** 2024-03-04

**Authors:** Aimin Zhang, Lei Li, Xuewen Xie, Ali Chai, Yanxia Shi, Dan Xing, Zhiguo Yu, Baoju Li

**Affiliations:** 1 Plant Protection College, Shenyang Agricultural University, Shenyang 110866, China; zhangaimin99@126.com; 2 Institute of Vegetables and Flowers, Chinese Academy of Agricultural Sciences, Beijing 100081, China; lilei01@caas.cn (L.L.); xiexuewen@caas.cn (X.X.); chaiali@caas.cn (A.C.); shiyanxia@caas.cn (Y.S.); 3 Institute of Pepper, Guizhou Academy of Agriculture Science, Guiyang 550025, China

**Keywords:** pepper anthracnose, typical symptoms, morphological identification, polygenic phylogenetic analysis

## Abstract

Anthracnose of pepper is a significant disease caused by *Colletotrichum* spp. In 2017 and 2021, 296 isolates were obtained from 69 disease samples. Through morphological analysis, pathogenicity detection, and polygenic phylogenetic analysis, the above strains were attributed to 10 species: *C. scovillei*, *C. fructicola*, *C. karstii*, *C. truncatum*, *C. gloeosporioides*, *C. kahawae*, *C. boninense*, *C. nymphaeae*, *C. plurivorum*, and *C. nigrum*. *C. scovillei* had the most strains (150), accounting for 51.02% of the total isolates; *C. fructicola* came in second (72 isolates), accounting for 24.49%. Regarding regional distribution, Zunyi City has the highest concentration of strains—92 strains total, or 34.18%—across seven species. Notably, this investigation showed that *C. nymphaeae* infected pepper fruit for the first time in China. Genetic diversity analysis showed that *C. fructicola* could be divided into seven haplotypes, and the population in each region had apparent genetic differentiation. However, the genetic distance between each population was not significantly related to geographical distance. Neutral detection and nucleotide mismatch analysis showed that *C. fructicola* might have undergone population expansion.

## 1. Introduction

Pepper (*Capsicum annuum* L.) is an important vegetable crop. There are about 2.1 million hectares of pepper planting area in China, and Guizhou Province has exceeded 300 thousand hectares [1], ranking first in China. In 2019, the chili pepper industry was listed as one of the “Twelve Characteristic Agricultural Industries” in Guizhou. The wider development of this industry has led to the annual expansion of the planting area. Factors such as limited cultivated land area have led to the increasingly prominent phenomenon of pepper continuous cropping, and the occurrence of soil-borne diseases, especially pepper anthracnose, has become increasingly severe.

Anthracnose is one of the principal plant diseases, the pathogen belonging to the genus *Colletotrichum* of the Coelomycetes of Deuteromycotina, and the fungi of this genus have a wide host range and often cause anthracnose of various crops [2]. The classification of *Colletotrichum* is complex because the genus has extremely complex genetic variation characteristics. Currently, the genus includes at least 14 species complexes and 13 singleton species [3]. Taxonomic research has evolved from morphological identification to a comprehensive evaluation system that includes morphological identification, pathogenicity detection, physiological characteristics, multi-gene joint tree-building analysis, and other indicators. Morphological identification mainly adopts the methods of Cai [4] and Sutton [5]. Detection indexes include colony culture morphology and growth rate, morphology and size of conidia and appressoria, presence and morphology of setae and sclerotia, etc. For phylogenetic analysis, at least 22 genes—including internal transcribed space (ITS), β-tubulin 2 (*TUB2*), actin (*ACT*), calmodulin (*CAL*), glyceraldehyde-3-phosphate dehydrogenase (*GAPDH*), histone3 (*HIS3*), chitin synthase 1 (*CHS-1*), histidinol dehydrogenase (*HIS4*), glutamine synthetase (*GS*), elongation factor 1α (*EF1α*), portions of the single-copy manganese superoxide dismutase (*SOD* 2), the 3′ end of the apurinic DNA lyase 2 (*Apn2*), the combined 5′ end of the mating-type idiomorph MAT1, the intergenic region of Apn2 and Mat1-2-1 (*ApMat*), and others—were used for molecular identification of *Colletotrichum* spp. [3]. In specific studies, the types and numbers of genes used by different scholars vary [4,6,7], with the top seven genes being used more frequently. Damm et al. [6,8,9,10,11,12] have used the above genes in studying multiple composite populations of *Colletotrichum*, as have Yang et al. [13], Liu et al. [14], and Diao et al. [15] when researching the pathogen of different plant anthracnose.

According to statistics, there were at least 31 species of pathogens causing pepper anthracnose [3,14,16,17], identified by multi-locus phylogeny, which were distributed in seven species complexes: *C. acutatum* complex (8), *C. boninense* complex (3), *C. gloeosporioides* complex (12), *C. magnum* complex (2), *C. orchidearum* complex (2), *C. truncatum* complex (1), *C. spaethianum* complex (1), and two singleton species, *C. coccodes* and *C. nigrum*. As many as 22 species have been reported in China [14,15,18]; among them, *C. fructicola*, *C. gloeosporioides*, *C. scovillei*, and *C. truncatum* were common strains. Effectively preventing and controlling anthracnose has become an important task. Currently, anthracnose prevention and control methods include using resistant varieties [19,20] and chemical agents [21,22] and identifying and screening biocontrol microorganisms [23,24]. Most of the above techniques target one or several types of anthrax bacteria. However, the pathogenicity of different strains of pepper and their sensitivity to pesticides are different [25,26,27,28], which makes it difficult to prevent and control pepper anthracnose.

To clarify the occurrence, main pathogen species, and distribution of pepper anthracnose in the main pepper-producing areas of Guizhou province, the disease survey and collection of disease samples were conducted in eight cities (prefectures). Pathogen isolation and purification, pathogenicity determination, and strain identification were carried out to pave the way for the next step of prevention and control.

## 2. Results

### 2.1. Typical Symptoms of Pepper Anthracnose

**Figure 1:**
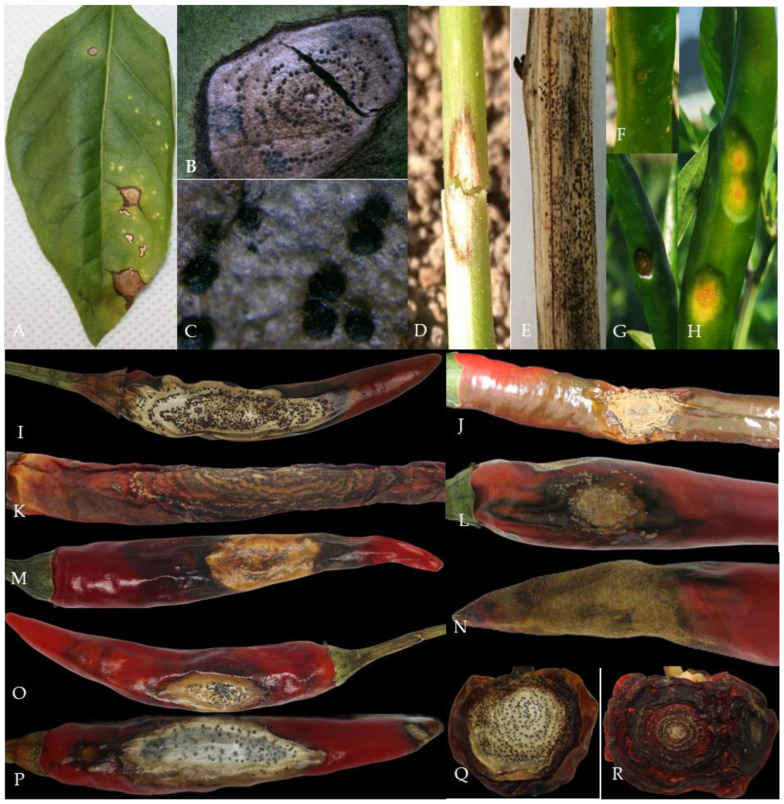
Typical symptoms of pepper anthracnose. Notes: (**A**)—initial symptoms of leaf infection with *Colletotrichum* sp.; (**B**,**C**)—acervuli on leaf; (**D**)—initial symptoms of stem infection with *Colletotrichum* sp.; (**E**)—the later symptoms of stem infection with *Colletotrichum* sp.; (**F**–**H**)—initial symptoms of fruits infection with *Colletotrichum* sp.; (**I**–**R**)—different symptoms of fruits infection with *Colletotrichum* sp. in the later stage.

The survey found that pepper anthracnose could occur from seedling to harvest and infect stems, leaves, and fruits (Figure 1). The pepper seedlings in the cold bed nursery and the leaves and stems from the field transplanting to the fruiting period (April to May) were susceptible to infection (Figure 1A–E). The pepper fruit from the green ripening period to the harvest period (mid-late July to late September) was the most seriously affected, which could easily cause severe economic losses.

At the initial stage of infection, the leaves and stems showed dark green water-immersed spots (Figure 1A,D), and at the later stage, the centers of the disease spots were brown or gray-white, commonly with black acervuli, either scattered or in concentric rings (Figure 1B,C,E).

At the early stage of the disease, it appeared in the pepper fruit as a round disease spot, usually in the form of water immersion (Figure 1F–H), and at the later stage, it formed a concave or non-concave disease spot. The disease spot had obvious or non-obvious concentric rings, and the color of the disease spot was brown, gray-white, or black. When the humidity was high, it was easy to produce an orange-red conidia pile; when the air was dry, the black acervuli with or without setae could be seen (Figure 1I–R), or the fruit peel was membranous and cracked.

### 2.2. Pathogen Morphological Characteristics

**Figure 2:**
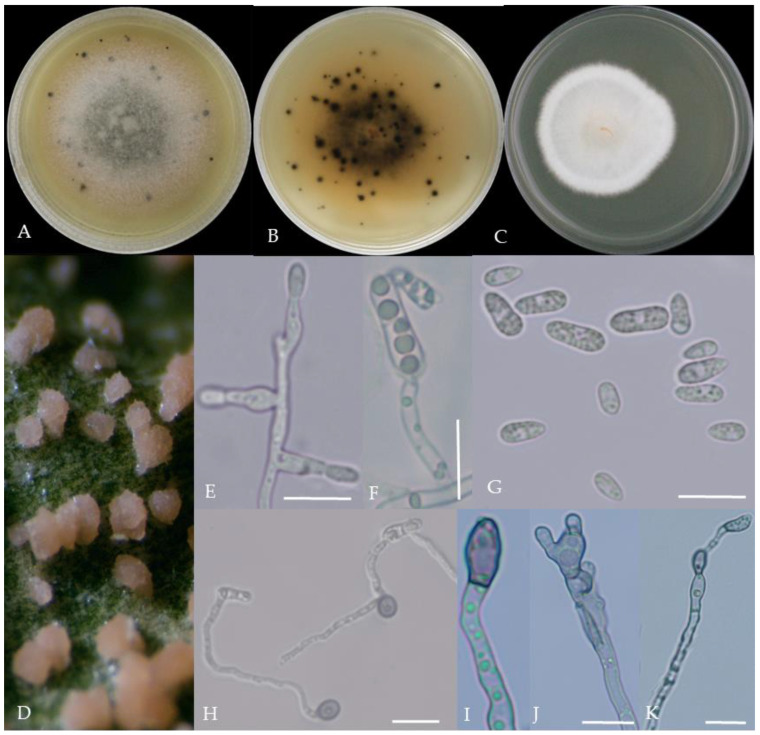
Morphological characteristics of Group 1 (*C. scovillei*). Notes: (**A**–**C**)—colonies on PDA above and below; (**D**)—conidia piles on the host; (**E**,**F**)—conidiophore; (**G**)—conidia; (**H**)—conidia appressorium; (**I**–**K**)—hyphal appressorium. Scale bars are 10 μm, the same as below.

**Figure 3:**
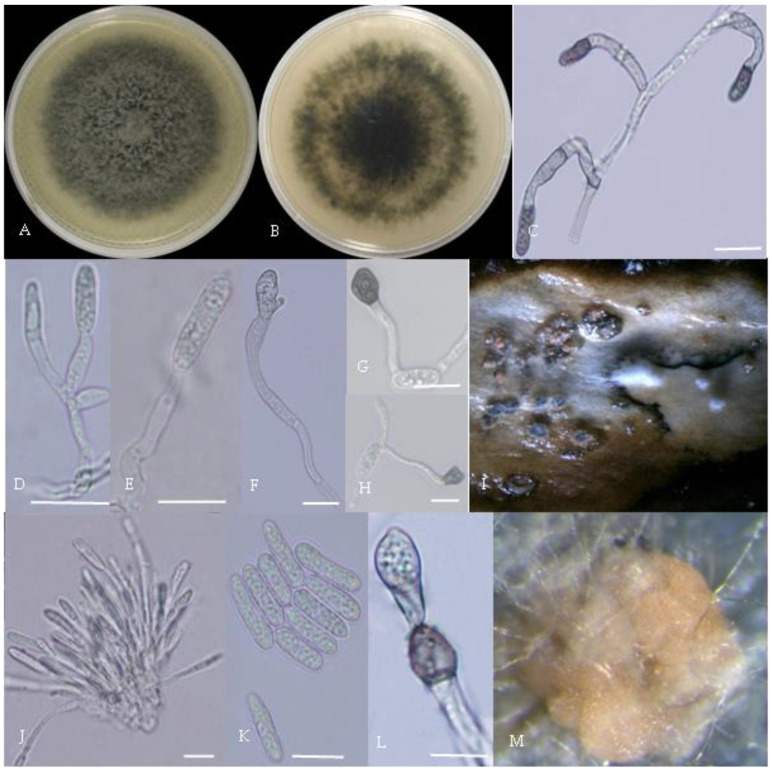
Morphological characteristics of Group 2 (*C. fructicola*). Notes: (**A**,**B**)—front and back of colony; (**C**,**F**,**L**)—hyphal appressorium; (**D**,**E**)—conidial peduncle and conidial disk; (**G**,**H**)—conidia appressorium; (**I**,**J**)—conidia disk on the host; (**K**)—conidia; (**M**)—conidia pile.

**Figure 4:**
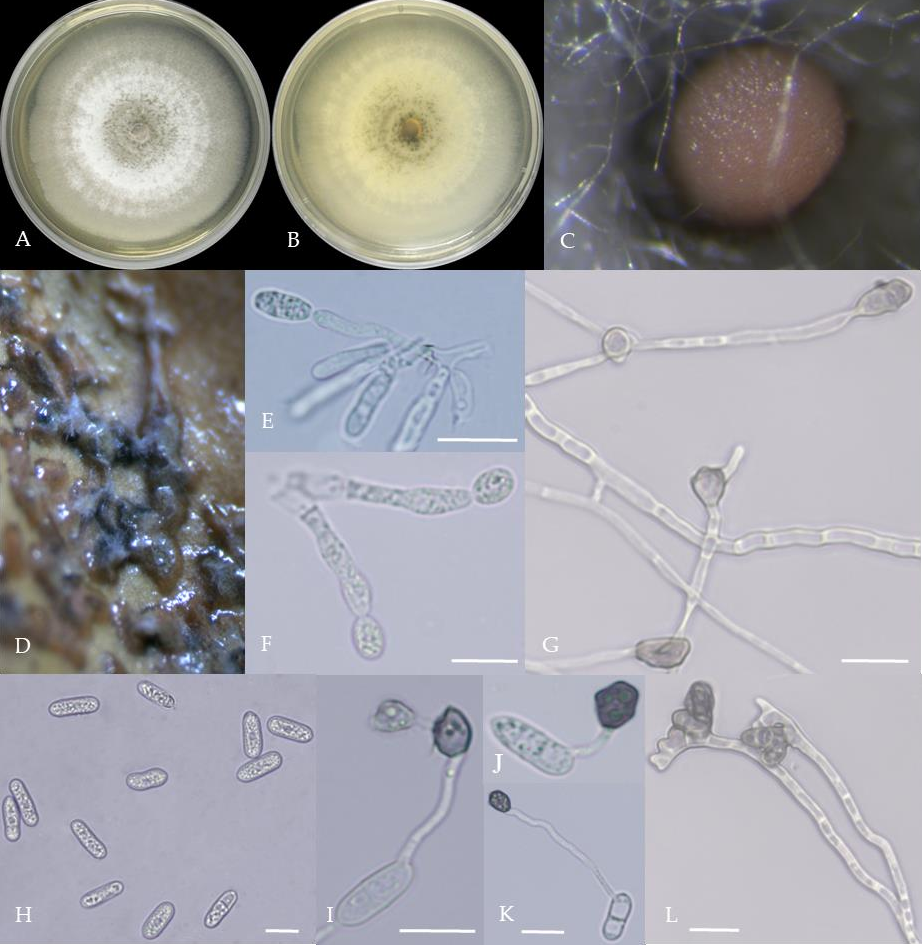
Morphological characteristics of Group 3 (*C. karstii*). Notes: (**A**,**B**)—colony front and back; (**C**)—conidia pile on PDA; (**D**)—conidia pile on the host; (**E**,**F**)—conidiophore; (**H**)—conidia; (**I**–**K**)—conidia appressorium; (**G**,**L**)—hyphal appressorium.

**Figure 5:**
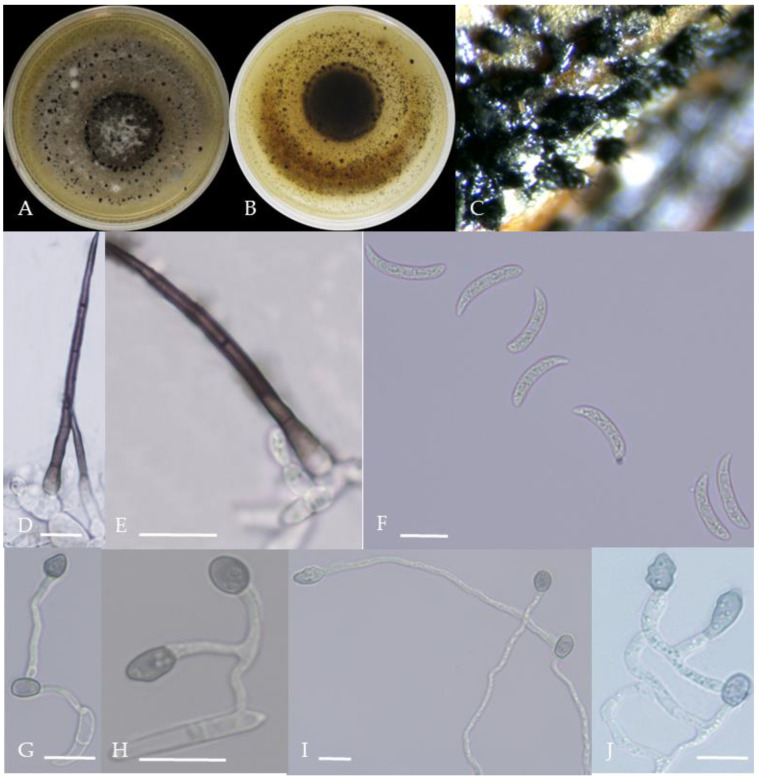
Morphological characteristics of Group 4 (*C. truncatum*). Notes: (**A**,**B**)—colony front and back; (**C**)—the conidia pile on the host; (**D**,**E**)—bristles; (**F**)—conidia; (**G**,**H**)—conidia appressorium; (**I**,**J**)—hyphal appressorium.

**Figure 6:**
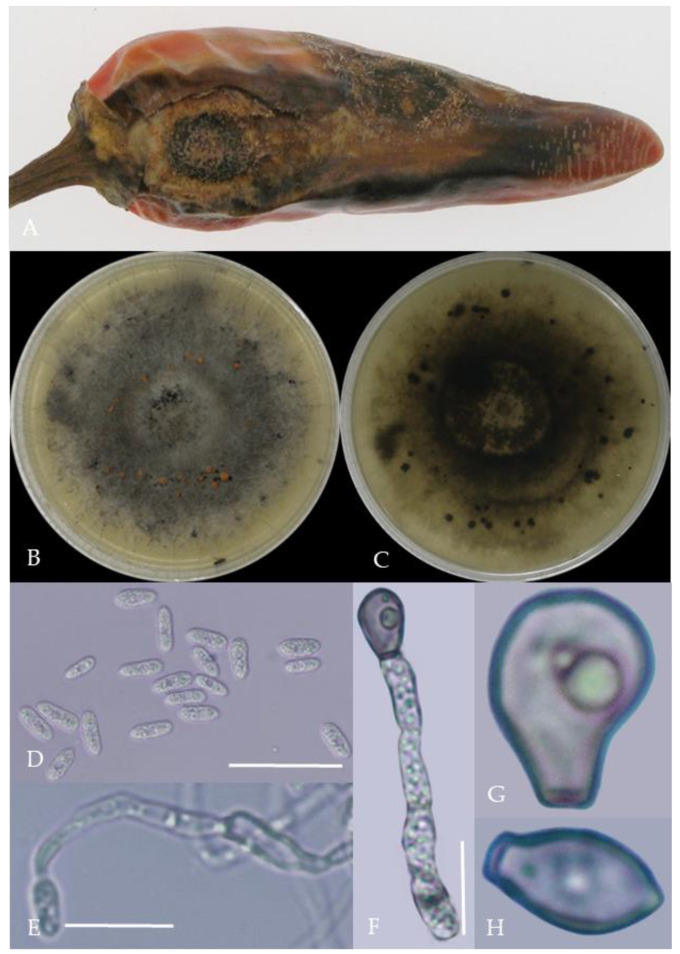
Morphological characteristics of Group 5 (*C. gloeosporioides*). Notes: (**A**)—disease spots on the host caused by *C. gloeosporioides*; (**B**,**C**)—front and back of colony; (**D**)—conidia; (**E**–**H**)—hyphal appressorium.

**Figure 7:**
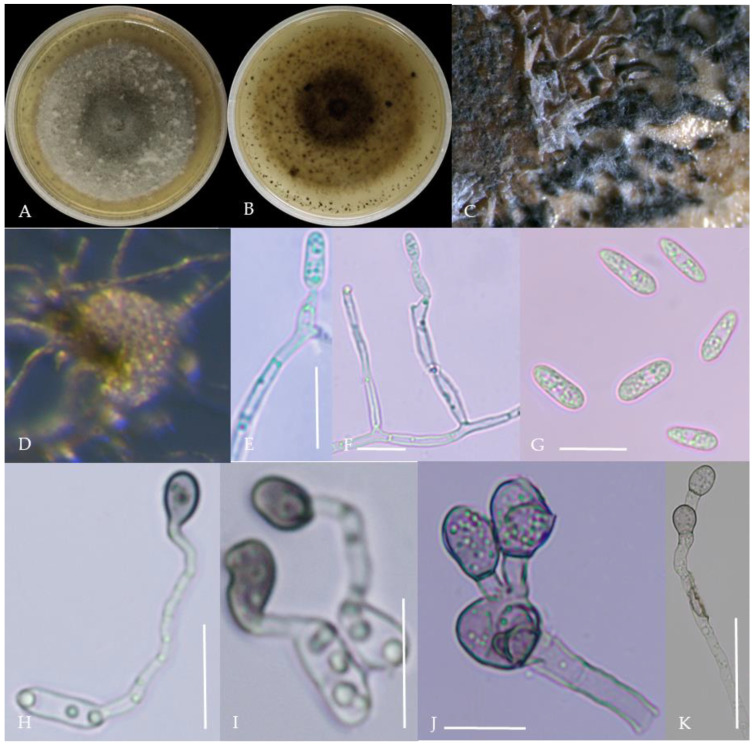
Morphological characteristics of Group 6 (*C. kahawae*). Notes: (**A**,**B**)—colony above and below; (**C**)—conidia pile on the host; (**D**)—conidia pile on WA; (**E**,**F**)—conidiophore; (**G**)—conidia; (**H**,**I**)—conidia appressorium; (**J**,**K**)—hyphal appressorium.

**Figure 8:**
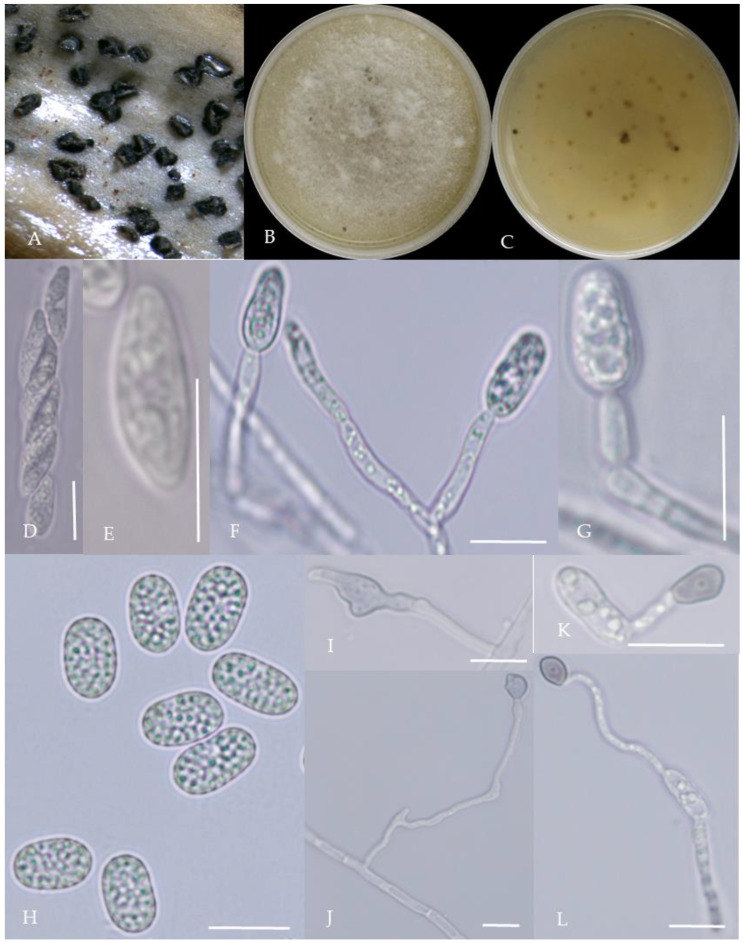
Morphological characteristics of Group 7 (*C. boninense*). Notes: (**A**)—conidia disk on the host; (**B**,**C**)—above and below of colony; (**D**,**E**)—sporangium and ascospore; (**F**,**G**)—conidiophore; (**H**)—conidia; (**I**,**J**)—hyphal appressorium; (**K**,**L**)—conidia appressorium.

**Figure 9:**
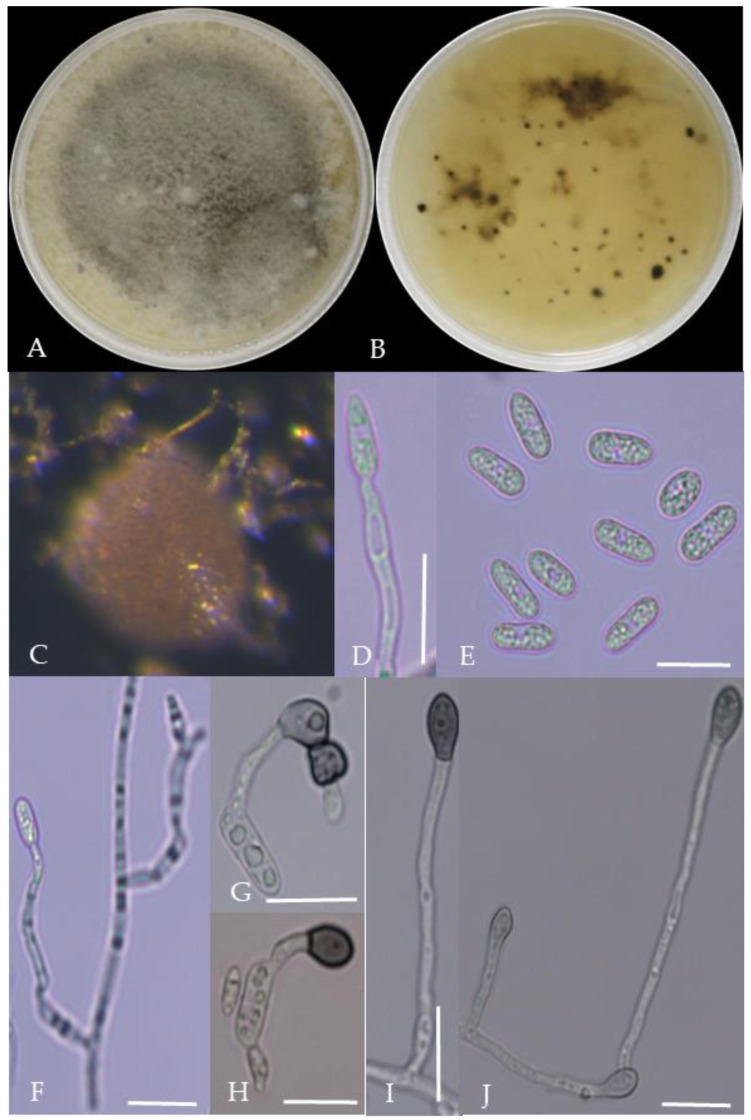
Morphological characteristics of Group 8 (*C. nymphaeae*). Notes: (**A**,**B**)—colony above and below; (**C**)—conidia pile on WA; (**D**,**F**)—conidiophore; (**E**)—conidia; (**G**,**H**)—conidia appressorium; (**I**,**J**)—hyphal appressorium.

**Figure 10:**
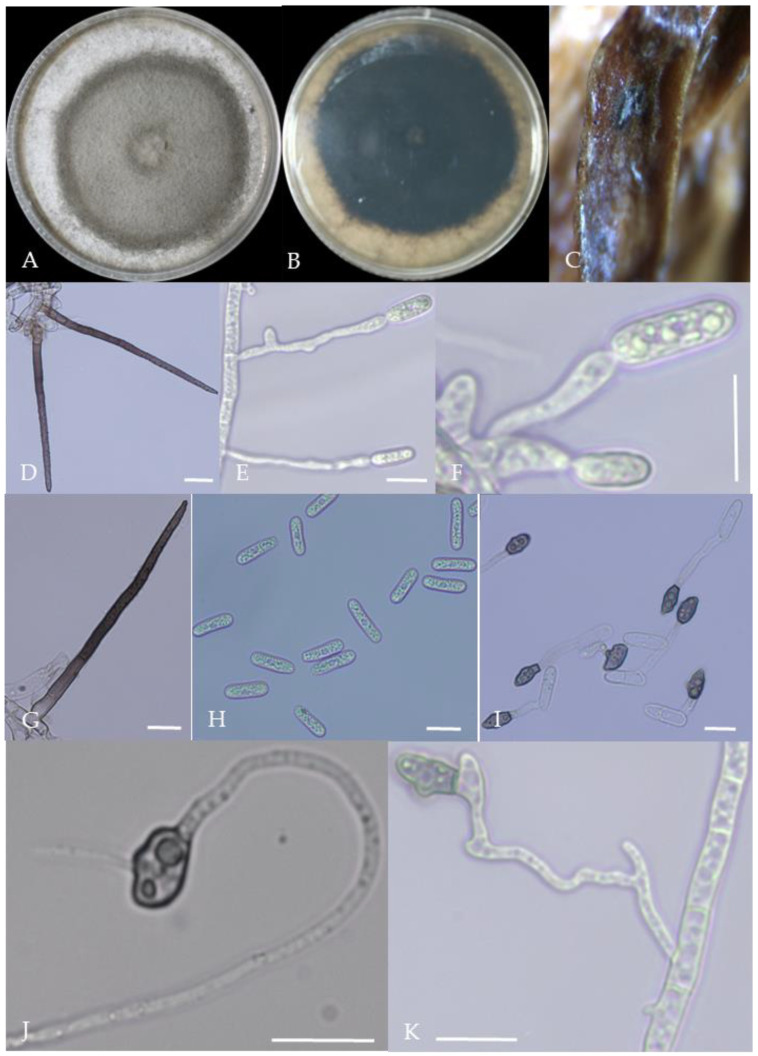
Morphological characteristics of Group 9 (*C. plurivorum*). Notes: (**A**,**B**)—colony above and below; (**C**)—disease spot on the host; (**D**,**G**)—bristles; (**E**,**F**)—conidiophore; (**H**)—conidia; (**I**)—conidia appressorium; (**J**,**K**)—hyphal appressorium.

**Figure 11:**
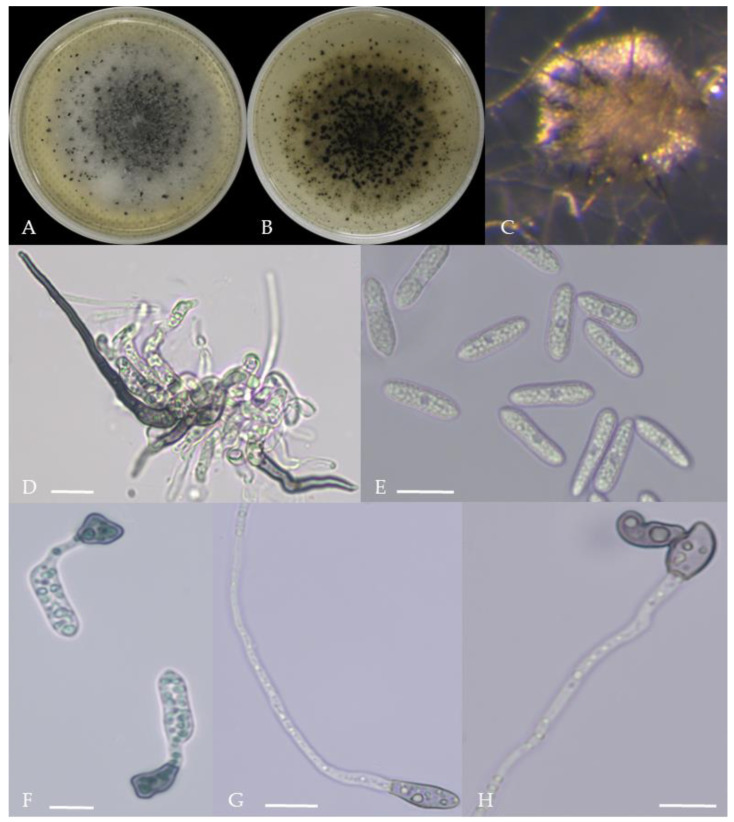
Morphological characteristics of Group 10 (*C. nigrum*). Notes: (**A**,**B**)—colony above and below; (**C**)—conidia pile on WA; (**D**)—seta; (**E**)—conidia; (**F**)—conidia appressorium; (**G**,**H**)—hyphal appressorium.

From the perspective of colony morphology (Table 1, Figure 2, Figure 3, Figure 4, Figure 5, Figure 6, Figure 7, Figure 8, Figure 9, Figure 10 and Figure 11), the isolate could be divided into 10 groups. Group 1 was significantly different from other colonies. After seven days of colony growth, these became orange-red, milky white, or gray, and villiform; a large number of cylindrical to oval conidia were produced on the mycelium, and about 1 month later, they produced black sclerotia.

Gray colonies included seven groups. Group 2 colonies were gray, with lush and fluffy hyphae, grayish green on the back, with no sclerotia and long cylindrical conidia; orange-red conidia piles could be produced on WA media. Group 4 was dark gray to light gray, with sparse hyphae, producing many scattered black sclerotia and crescent-shaped conidia, with one end rounded and one tapered. The colonies of Group 5 were light gray and fluffy, with orange-red conidia piles and black sclerotia produced in the later stage, and the conidia were cylindrical to oval in shape. The colonies of Group 6 were light gray, with dense hyphae that were like a tapestry, and the back of the colonies were brown; in the later stage, scattered black small sclerotia and conidia piles formed on the WA, and the conidia were cylindrical to oval in shape. Group 8 was light gray, with dense tapestry-like hyphae, milky white to light yellow on the back, scattered with a small number of sclerotia; orange conidia piles were produced on the WA, and the conidia were nearly round or cylindrical. Group 9 colonies were dark gray in the middle, with milky white edges, dense tapestry-shaped hyphae, dark gray to black on the back, and long cylindrical conidia. The colonies of Group 10 were gray, with a darker color in the middle; the hyphae were luxuriant and fluffy, with a large number of black sclerotia scattered; orange conidia piles produced on the WA, and brown seta were visible; the conidia were obtusely rounded at both ends, forming a long cylindrical shape, or one end was obtusely rounded and the other end was gradually pointed, forming a stick shape.

There were two groups with white colonies. Group 3 had white colonies with apparent concentric rings, and in the later stage, gray sclerotia was produced in the center of the colonies, while the back of the colonies was light yellow; orange-red conidia piles produced on WA medium, and the conidia were cylindrical-shape. Group 7 colonies were white, with dense tapestry-like hyphae, pale yellow on the back, producing gray sclerotia and cylindrical conidia; sometimes sexual asci and ascospores could be seen, and the ascus contained 6–8 ascospores, which were spindle-shaped.

### 2.3. Pathogenicity Test

**Figure 12:**
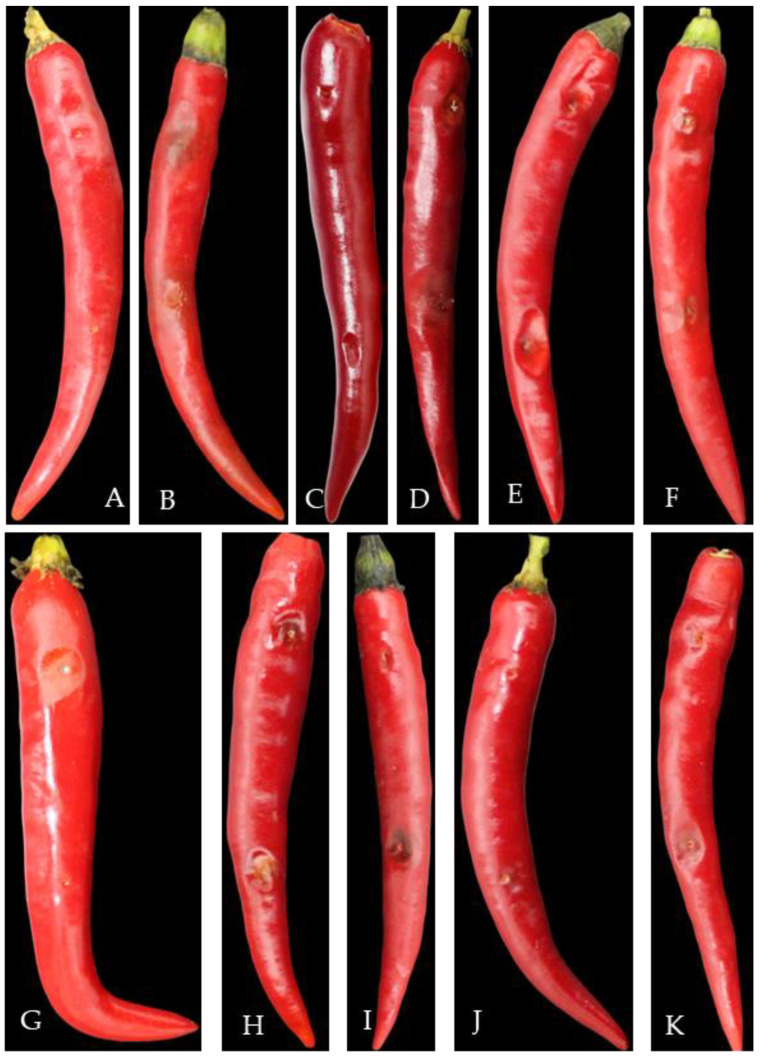
Pathogenicity test of pepper anthracnose pathogens. Note: (**A**)—CK, (**B**)—*C. fructicola*, (**C**)—*C. gloeoporioides*, (**D**)—*C. nymphaeae*, (**E**)—*C. scovillei*, (**F**)—*C. kahawae*, (**G**)—*C. boninense*, (**H**)—*C. nigrum*, (**I**)—*C. plurivorum*, (**J**)—*C. karstii*, (**K**)—*C. truncatum*.

Seven days after inoculation with different pathogens, pepper fruit showed different symptoms of disease spots, similar to the symptoms of natural disease in the field, and no disease symptoms were observed in the control treatment (Figure 12). The pathogens isolated from diseased fruits had the same morphological characteristics as the inoculated pathogens.

### 2.4. Polygenic Phylogenetic Analysis

**Figure 13:**
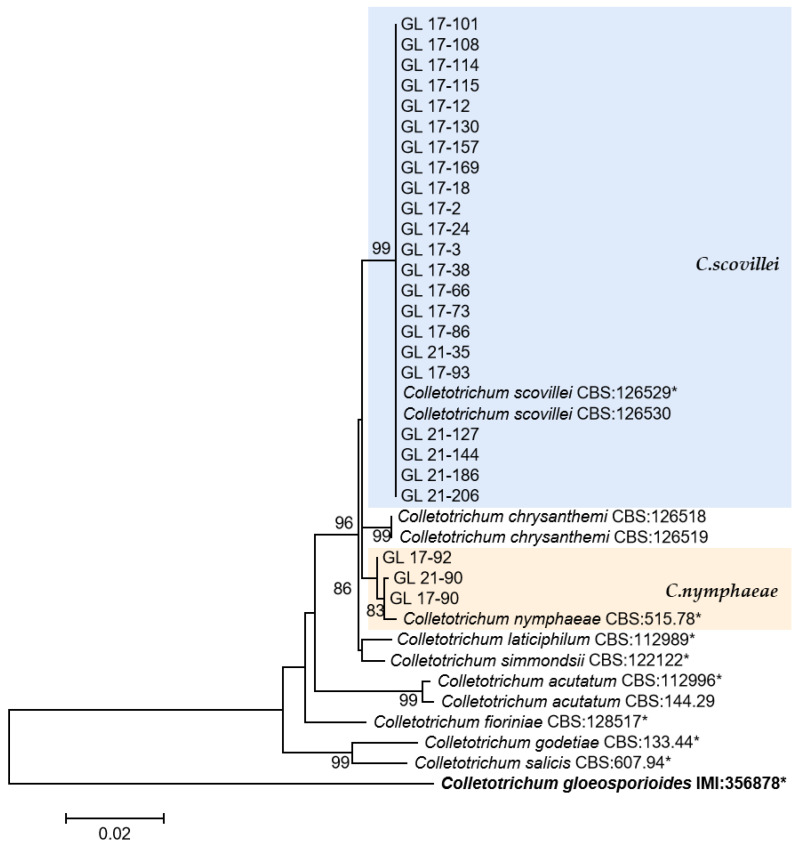
The *C. acutatum* complex. Notes: This development tree was constructed by the Maximum Likelihood method in MEGA 6.06 software after six genes, such as ITS, ACT, CHS-1, GADPH, TUB2, and HIS 3, were compared and spliced by SequenceMatrix. The number on the branch node represents the support rate obtained by Bootstrap replication calculation 1000 times. The sample strains in the figure were only representative strains in the isolated strains, and the strains with * were type, ex-type, or ex-epitype strains. Bold represents the outgroup.

**Figure 14:**
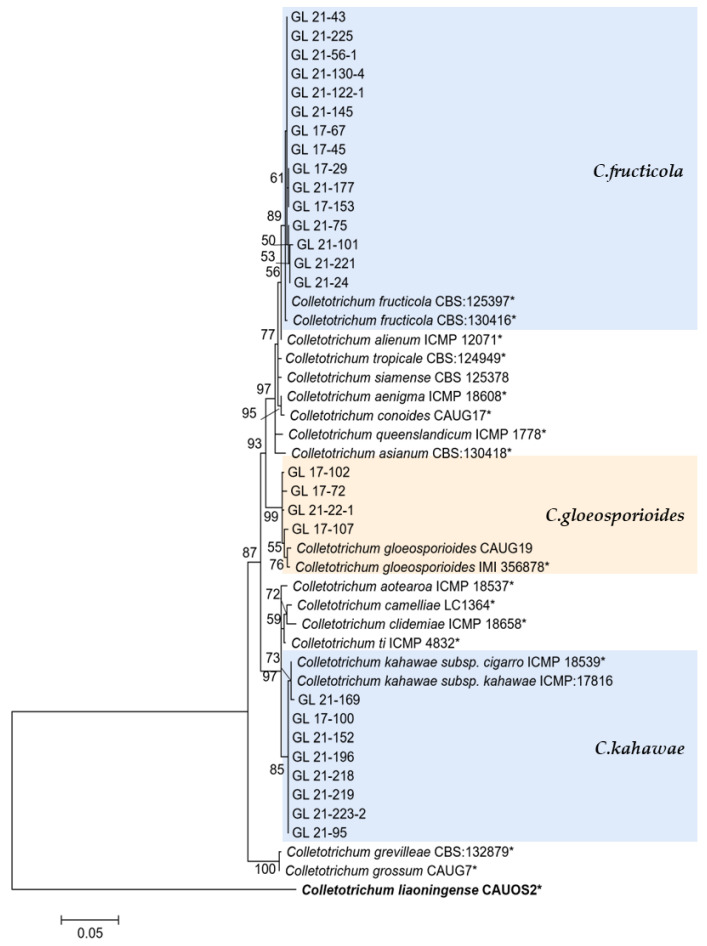
The *C. gloeosporioides* complex. Notes: The strains with “*” were ex-type or ex-epitype cultures. Bold represents the outgroup.

**Figure 15:**
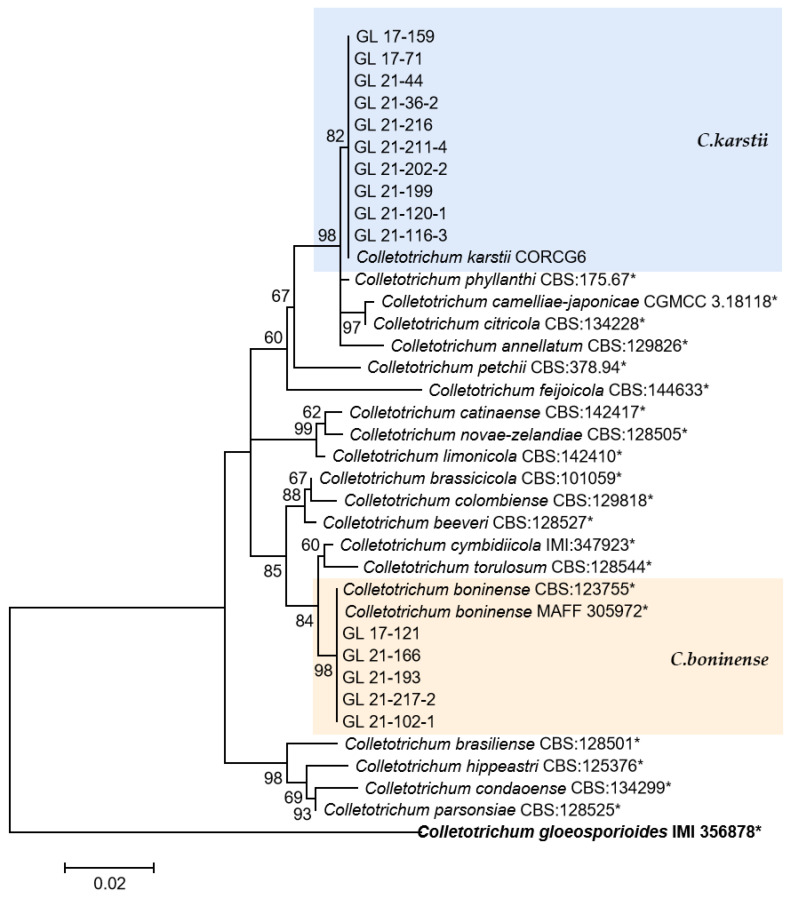
The *C. boninense* complex. Notes: The strains with “*” were ex-type or ex-epitype cultures. Bold represents the outgroup.

**Figure 16:**
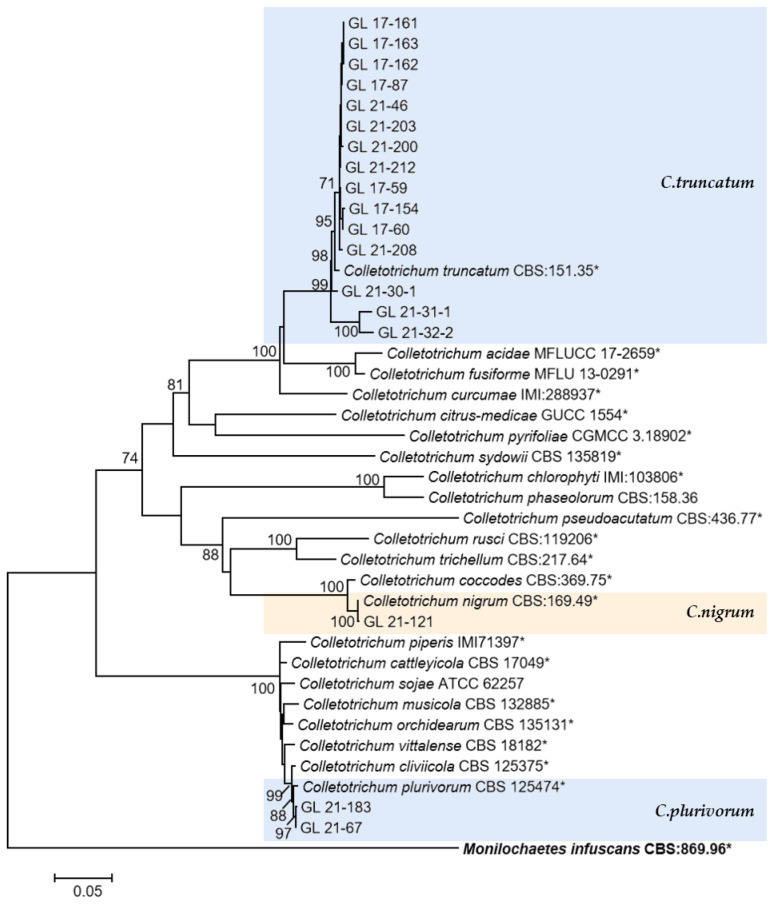
The *C. truncatum* complex, *C. orchidearum* complex, and the singleton species. Notes: The strains with “*” were ex-type or ex-epitype cultures. Bold represents the outgroup.

The multi-locus phylogenetic analysis based on five to six genes (Table 2, Table 3, Table 4 and Table 5) showed that 296 isolates belonged to 10 species (Figure 13, Figure 14, Figure 15 and Figure 16), of which 150 isolates were identified as *C. scovillei*, accounting for 51.02% of the total number of strains, followed by *C. fructicola*, *C. karstii*, *C. truncatum*, *C. gloeosporioides*, *C. kahawae*, and *C. boninense*. They numbered 74, 19, 17, 12, 10, and 8 isolates, respectively, accounting for 25.17%, 6.46%, 5.78%, 4.08%, 3.40%, and 2.72% of the total isolates. In addition, there were 3, 2, and 1 strains of *C. nymphaeae*, *C. plurivorum*, and *C. nigrum*, respectively.

This was the first report of *C. nymphaeae*-caused anthracnose in chili peppers in China. Two isolates were isolated from Huangping County of Qiandongnan State in 2017 and one isolate was from Ziyun County of Anshun City in 2021. Whether there is a risk of diffusion of this pathogen in chili peppers remains to be studied.

### 2.5. Geographical Distribution of Pathogens

**Figure 17:**
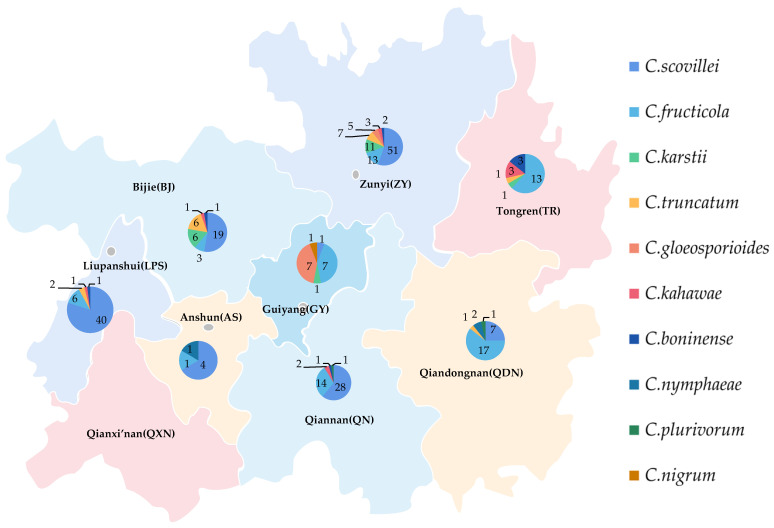
Geographical distribution ten species of *Colletotrichum* spp. in Guizhou.

There was a significant disparity in the strains’ number of different species obtained from different times and locations (Figure 17). In total, 103 strains of *C. scovillei* were isolated in 2017, and 47 were isolated in 2021, ranking first in number of all species, which should make this the most important pathogen of pepper anthracnose in Guizhou Province. The number of strains of *C. fructicola* was 7 in 2017 and 67 in 2021; the number of strains identified as *C. karstii* was 2 and 17, respectively. The increasing number of strains of the above two species might indicate that the types of primary pathogens would change.

The distribution proportions of isolates’ number of different species in various regions were quite distinct (Figure 17). Among the eight regions, 91 isolates of the primary pathogens *C. scovillei* were isolated in Zunyi (ZY) and Liupanshui (LPS), accounting for 60.67% of the isolates of this species, followed by Qiannan (QN) and Bijie (BJ), with 28 and 19 isolates, respectively. The pathogens isolated in ZY included seven species, 92 isolates in total, accounting for 31.08% of the isolates. They were followed by LPS, QN, and BJ, accounting for 16.89% (five species), 15.88% (five species), and 12.16% (six species), respectively. The number of isolates and species from Anshun (AS) was the least, at six and three, respectively. The least number of species was of *C. nigrum*, whose only isolate was from Guiyang (GY).

### 2.6. Genetic Diversity of C. scovillei and C. fructicola

Polymorphism analysis was conducted on six genes of the top two species of isolate quantity, *C. scovillei* and *C. fructicola*. All six genes of 150 *C. scovillei* strains had no mutation sites. Therefore, no further analysis was conducted on this species.

**Figure 18:**
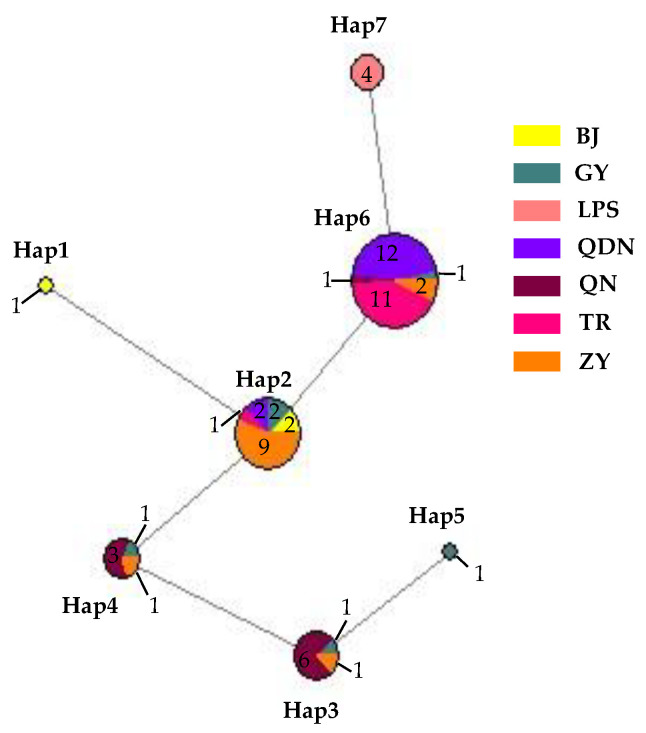
Network diagram and geographical distribution of *C. fructicola*.

Nucleotide composition analysis of ITS-ACT-CHS1-GAPDH-TUB2-HIS3 from 62 isolates of *C. fructicola* showed a G+C content of 0.559, with seven sites with alignment gaps or missing data and a total of six polymorphic sites, including three parsimony informative sites and three singleton variable sites. These polymorphic sites produced a total of seven haplotypes (Hd = 0.7277) (Figure 18), with the highest number being haplotype 6 (abbreviated as Hap 6, the same below). It had 27 isolates, accounting for 43.55% of the total, with 12 isolates in QDN and 11 isolates in TR, which was the leading distribution area; in addition, ZY had two isolates, and GY and QN each had one isolate. The second was Hap 2, with a total of 16 isolates, accounting for 25.80% of the total number of isolates, mainly distributed in ZY (9); in addition, there were two each in GY, BJ, and QDN, and one isolate in TR, respectively. Hap 1 and Hap 5 had one isolate isolated from BJ and GY, respectively. From the distribution of different haplotypes in various regions, the number of GY isolates was small, but the haplotypes were the highest, including all haplotypes except for Hap 1 and Hap 7; next was ZY, which contains 4 haplotypes; and the least was LPS, which only has Hap 7, and this haplotype was only distributed here, not found in other regions.

The analysis results conducted using GeneAlex showed a positive correlation (Rxy = 0.004) between the genetic distance and the geographical distance of *C. fructicola*. However, the correlation was not significant (*p* = 0.440). That indicated a certain degree of genetic differentiation among the population in different regions of Guizhou Province, but that this differentiation was not significantly correlated with geographical distance.

**Figure 19:**
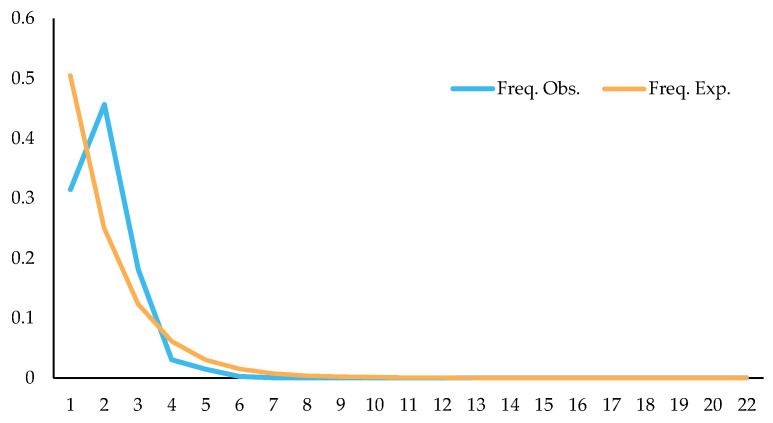
Mismatch distribution of *C. fructicola* population.

Studying changes in population dynamics by using neutral detection methods, results showed that Tajima’s D = −0.5689 (*p* > 0.10), Fu and Li’s D = −1.5171 (*p* > 0.10), and Fu and Li’s F = −1.4243 (*p* > 0.10); through mismatch distribution analysis of splicing sequences, it was found that the expected values were roughly consistent with the observed values, and the observed values showed a single peak (Figure 19). The above detection results indicated that *C. fructicola* might have population expansion, which might be why the population has no clear genetic differentiation among some regions, and one of the reasons why the correlation between genetic distance and geographical distance was not significant.

The Fst value (Table 6) could be used to preliminarily analyze the genetic differentiation relationship of the *C. fructicola* populations among different regions. Analysis results demonstrated that there was minimal genetic differentiation in the *C. fructicola* population of BJ and ZY, GY and ZY, LPS and QDN, and QDN and TR (0.05 < Fst < 0.15), while there was no genetic differentiation between LPS and TR (Fst = 0). Meanwhile, the Fst between other populations was greater than 0.25, which indicates significant genetic differentiation among the *C. fructicola* populations between these regions.

## 3. Discussion

This study aimed to understand the main hazards and typical symptoms of pepper anthracnose and identify the types and distribution of pathogens that cause pepper anthracnose in Guizhou Province. Field observations found that this disease could occur from the seedling stage to the fruit ripening period. At the seedling stage, it mainly infected leaves, and it became increasingly severe from the green ripening to the complete ripening stage of fruits, with the occasional occurrence of stem infections. At the early stage of the disease, various tissues often exhibited light brown water-soaked lesions. At the later stage of the disease, most leaves and stems showed spherical black acervuli with seta, while the fruit symptoms were relatively diverse. Some lesions were sunken, and some were characterized by exfoliation of the stratum corneum without significant sunkenness. On some could be seen scattered or ring-shaped black acervuli, and on others orange-red conidial masses; the above symptoms may be related to the climatic environment [29]. Some disease spots might have compound infections and complex symptoms. In short, it was difficult to distinguish the types of pathogens from disease symptoms, and scientific methods were needed to identify them.

Through morphological and phylogenetic analysis, and pathogenicity identification, 296 strains of *Colletotrichum* were identified as *C. scovillei* (150 strains, 51.02%), *C. fructicola* (74 strains, 25.17%), *C. karstii* (19 strains, 6.46%), *C. truncatum* (17 strains, 5.78%), *C. gloeosporioides* (12 strains, 4.08%), *C. kahawae* (10 strains, 3.40%), *C. boninense* (8 strains, 2.72%), *C. nymphaeae* (3 strains, 1.02%), *C. plurivorum* (2 strains, 0.68%) and *C. nigrum* (1 strain, 0.34%), respectively.

Morphological identification is the most fundamental aspect of fungal species identification. Cai et al. [4] suggested that a mycelial disc (about 4 mm) be taken from the edge of a five-day-old colony with vigorous activity and inoculated in PDA plates at 20 °C, 25 °C, and 30 °C under constant fluorescence light to observe the growth rate and morphological characteristics of the *Colletotrichum* fungi. However, Damm et al. [8,9,10,11,12] still used their method to observe the features of the colony and characteristic structures. They used the SNA and OA cultures to incubate at 20 °C under near-UV light with a 12 h photoperiod for 10 d. Torres-Calzada et al. [30] placed the mycelial plugs onto the PDA dishes and incubated them at 25 °C for seven days to describe the colonies’ growth rate, color, shape, and conidial morphology of *C. truncatum*. By this token, there is still no unified standard for the cultural conditions used for the morphological identification of *Colletotrichum*. In the present study, we used PDA culture medium; the colony growth diameter and spore production were measured after seven days of natural light cultivation at 28 °C. After 30 days of cultivation, we observed whether the sclerotia and spore production structure were produced. The isolates had significant morphological differences, and preliminary grouping could be conducted based on morphological characteristics. Still, the results differed from those of Liu et al. [14] regarding growth rate, conidia, and appressorium morphology. Different cultural conditions probably caused this. The morphology of pathogens was relatively sensitive to environmental conditions. Therefore, Cai et al. [4] believed that many problems in species identification could not be solved entirely solely through physiology, but that it was possible to establish specification boundaries for existing names and introduce new specifications through the polymorphic approach.

From the analysis of morphological characteristics, most of the colonies of *C. scovillei* were orange-red or light gray, villous, and had significant differences in morphology from other *Colletotrichum* species, making them easier to distinguish. However, in this study, one isolate that did not produce orange-red pigment or conidia and had a slow growth rate was identified as *C. scovillei* by phylogenetic analysis. Pathogenicity testing showed that this strain could cause anthracnose in green and red ripe fruits of chili peppers; when the humidity was high, orange-red conidia piles were produced. The strain obtained from re-isolation was similar to other *C. scovillei* isolates. The reason for the variation of this isolate was still unclear. The setae of *C. fructicola* were rarely found. Yang [18] found no seta in the strain isolated from chili peppers. Liu et al. [31] only found one bristle in *C. fructicola* isolated from *Camellia*. In this study, setae were found on one conidial disk of WA medium, and their morphological characteristics were consistent with Liu et al.’s description.

Multi-gene phylogenetic analysis is one of the critical research contents of *Colletotrichum* species identification, but different research teams use different genes. Crouch et al. used ITS, *HMG*, *Apn2*, *Mat1-2*, and *SOD2* in 2006 and 2009 to identify various gramineous plant anthracnose pathogens and conducted phylogenetic and population genetic differences analysis on *Colletotrichum cereale* in different grassland populations. The Cai research group [13,15,32,33] conducted a classification analysis of *Colletotrichum* spp. They were isolated from different plants using ITS, *ACT*, *GAPDH*, *HIS3*, *CHS-1*, *TUB2*, *CAL*, and *GS*. Damm et al. [6,8,9,10,11,12,34] used the same genes (except *GS*) to comprehensively descript and identify *Colletotrichum* spp., which include *Colletotrichum* with curved conidia, *C. acutatum*, *C. destructivum*, *C. dracaenophilum*, *C. magnum*, and *C. orchidearum* species complexes, as well as *C. eriobotryae* sp. nov. and *C. nymphaeae* isolated from loquat fruit. This study utilized six common genes from the Cai research group and Damm et al. to conduct multi-locus phylogenetic analysis on isolates and identified 296 isolates as 10 *Colletotrichum* species.

Research has found that distinguishing between *C. scovillei* and *C. guajavae* in the *C. acutatum* complex is challenging. The GAPDH sequence between the two species has 7 bp base difference, making it the sequence with the most significant difference; ITS only has a 1 bp base difference, while there was no difference between the other four gene sequences. This research result was similar to the research conclusion of Damm et al. [8]. *C. scovillei* might have been isolated initially from chili peppers by Nierenberg et al. [35], and BBA 70349 (PD 94/921-3) and PD 94/921-4 isolated from *Capsicum annuum* were identified as *C. acutatum* based on morphology and RAPD-PCR. In 2008, Than et al. [36] isolated Mj6 from *Capsicum annuum* and identified it as *C. acutatum* based on morphological observations and phylogenetic trees established by ITS and TUB; Damm et al. [8] found a multi-locus phylogenetic tree using six genes, corrected the above three strains to be *C. scovillei*, and used one of them as an ex-type strain. Subsequently, Kanto et al. [37], Liu et al. [14], and Diao et al. [15] isolated *C. scovillei* from the anthracnose samples of *Capsicum* spp. In this study, *C. scovillei* accounted for 51.02% of the total isolates, suggesting that this species may be the primary pathogen causing pepper anthracnose in Guizhou Province.

In this study, except for *C. scovillei*, *C. nymphaeae* was the only other species from the *C. acutatum* species complex. The six gene sequences of this species had 2–7 bp differences from *C. scovillei*, respectively. This species has been reported to attack crops including strawberries [38], apples [39], citrus [40], tomatoes [41], and more. In China, diseases caused by *C. nymphaeae* infection have been found in grapevine [14], loquat [42], peach [43], walnut [44], tobacco [45], *Camellia oleifera* [46], et al. In 2016, this strain was isolated from chili peppers in Malaysia [47], and in this study, it was found for the first time that this species caused pepper anthracnose in China.

This study’s *C. gloeosporioides* species complex strains isolated from diseased chili peppers include *C. gloeosporioides*, *C. fructicola*, and *C. kahawae*. From the perspective of the phylogenetic tree structure, the distribution of these three species was similar to the research conclusion of Weir et al. [48], indicating that the six genes used in this study were reliable in the identification of *C. gloeosporioides* species complex isolates. There have been widespread reports of *C. gloeosporioides* infecting chili peppers, including in China [14,15,18], Malaysia [49], and India [50]. The *C. gloeosporioides* isolated in this study only accounted for 4.08% of the total isolates, and there were more isolates in 2017 (10), indicating that *C. gloeosporioides* might not be the main pathogen of chili anthracnose in Guizhou, and that its harm had a decreasing trend. *C. fructicola* was first discovered on coffee berries in Thailand, but it was later discovered that the strain had a very wide host and distribution range and had records of infecting chili peppers worldwide [51]. There were records of this species causing chili anthracnose in various chili planting areas in China [14,15,18]. In this study, given that a total of seven isolates were isolated in 2017, and 67 isolates were isolated in 2021, it was the second most abundant strain, so it was one of the main pathogens of pepper anthracnose in Guizhou. *C. kahawae* was initially isolated from coffee berries and later used to define *Colletotrichum* sp. in the same host. Weir et al. [48] divided this species into two subspecies based on their pathogenicity to coffee berries—*C. kahawae* subsp. kahawae could trigger Coffee Berry Disease (CBD) and *C. kahawae* subsp. ciggaro could not cause CBD. The former only infected African coffee berries, while the latter had a wide distribution and host range [52,53]. The two subspecies could be distinguished and identified through GS and ApMat. Cabral et al. [52] proposed upgrading the *C. kahawae* subsp. ciggaro to a species and naming it *C. ciggaro*. However, this study did not conduct GS and ApMat sequencing, so the two subspecies could not be completely distinguished in the phylogenetic tree. Thus, 10 isolates similar to the two species were temporarily classified as *C. kahawae*. Their accurate classification will be further studied. There were more strains (nine) isolated in 2021 of *C. kahawae*, and further research was needed to determine whether this species will rise to become the main pathogen of pepper anthracnose in Guizhou.

The *C. boninense* and *C. karstii* isolates belong to the *C. boninense* species complex. Among them, *C. boninense* was first isolated from *Crinum asiaticum* in the Bonin Islands of Japan and later found on diseased and healthy plants such as *Orchidaceae*, *Amaryllidaceae*, *Bigoniaceae*, *Podocarpaceae*, *Proteaceae*, *Solanaceae*, and *Theaceae*, indicating a wide range of hosts and diverse lifestyles [9]. In 2009, Tozze et al. [54] first reported that *C. boninense* caused pepper anthracnose. In China, Yang [18] first isolated one strain of this species from diseased pepper fruits in Duyun, Guizhou. In 2013, Diao reported for the first time that *C. boninense* was isolated and identified on chili peppers in Sichuan, China [55]. In this study, eight strains of the species were isolated and distributed in five cities and prefectures in Guizhou Province, indicating that the fungus might have epidemic risks. *C. karstii* was collected from *Vanda* sp. leaves in Luodian County, Guizhou Province, by Yang et al. in 2009 [13], named after the geological characteristics of the collection site—karst. It is the most widely distributed strain in the *C. boninense* complex, and its hosts include *Orchidaceae*, *Annonaceae*, *Area*, *Bombacaceae*, *Theaceae*, and *Solanaceae* [9]. Yang [18] isolated this strain from Anshun, Duyun, and Tongzi chili peppers in Guizhou Province. The 19 isolates of *C. karstii* in this study were distributed in four cities and states; among them, there were more strains (11 and 6) in Zunyi and Bijie, indicating a wide distribution range of this pathogen, with northern Guizhou as the main distribution area.

The only species of *Colletotrichum* with curved conidia isolated in this study was *C. truncatum*, which is hosted by over 460 plant species and has been reported to harm chili peppers in multiple countries and regions, and this species has been isolated and identified in most chili planting areas in China [15]. In this study, 17 isolates of this species were isolated. From the phylogenetic tree structure, there was a clear grouping between the *C. truncatum* isolates isolated from the diseased fruits of Bijie (GL 21-30-1, GL 21-31-1, and GL 21-32-2) and other isolates. From the six gene sequences, there were 37 variable sites among all isolates, among which there were 28 variable sites between Bijie isolates and others, including 24 parsimony informative sites and four singleton variable sites. The reason for these site changes and their impact on strain characteristics need further research. Additionally, the relationship analysis between genetic diversity and geographical distribution had yet to be conducted due to the limited number of isolated strains.

*C. plurivorum* belongs to the *C. orchidearum* species complex, isolated originally from Sichuan diseased chili fruit by Liu et al. [14] and named *C. sichuanensis*. It was later recognized as the homonymous species of *C. cliviicola* in Douanla-Meli et al.’s study [56], while Damm et al.’s study [12] identified them as two different species. The former has a wide host range, and the latter was named after its host *Clivia*, which *GAPDH*, *TUB2*, and *HIS3* sequences could distinguish. In the phylogenetic tree of this study, the support rate on the branches of *C. plurivorum* and *C. cliviicola* was 98, indicating a close phylogenetic relationship between the two species. From the six gene sequences, there was one mutated base for ITS and *CHS-1*, two for *GAPDH*, three for *ACT* and *TUB2*, and five mutated bases for HIS3, which was similar to the research conclusion of Damm et al. [12].

Halsted [57] reported on the New Jersey pepper anthracnose disease caused by *C. nigrum*. In 1896, it was reported that this species was the main causal agent of the American pepper anthracnose disease. Subsequent studies found that the fungus had a wide range of hosts and, like *C. coccodes*, could cause anthracnose in chili peppers and tomatoes, but that only *C. coccodes* could cause potato black spots. From the phylogenetic tree, the phylogenetic relationship between *C. coccodes* and *C. nigrum* was extremely close, consistent with the research results of Liu et al. [58], with a support rate of 100 on both species’ branches. ITS had no differential bases from the gene sequence perspective, while *ACT*, *CHS-1*, *GAPDH*, *TUB2*, and *HIS3* have 2, 3, 6, 8, and 11 differential bases, respectively. This differed from the report by Jayawardena et al. [3], which reported that the two species could be distinguished with ITS. This might be related to the different gene fragments used. In addition, regarding conidia morphology, *C. nigrum* had longer conidia and larger L/W values than *C. coccodes*. The conidia size of the isolates in this study was 9.41–17.45 × 3.14–4.31 μm; it was closer to the *C. nigrum* described by Liu et al. [58]. Therefore, the isolate was classified as *C. nigrum*.

## 4. Materials and Methods

### 4.1. Sample Collection, Pathogen Isolation and Purification

During the 2017 and 2021 Guizhou pepper industry censuses, 69 samples of fruits, leaves, and stems of pepper with anthracnose symptoms were collected from 44 locations in Guizhou Province by personnel related to pepper disease research. Using the stereoscopic microscope (Olympus SZX16, Olympus Corporation, Tokyo, Japan) and the optical microscope (Olympus CX31, Olympus Corporation, Tokyo, Japan), we observed the scabs and pathogen and took photos to record the samples infected with *Colletotrichum* spp.

Two methods were used to isolate and purify pathogens. If conidia had already been produced on tissues of pepper in nature, we used a sterile insect needle (2#) to pick up the conidia into sterile water, prepared a suspension of 1.0 × 10^4^ spores/mL of conidia, took 100 μL of the above suspension, uniformly spread with a stainless steel spreader (triangle End 16 mm, 5 mm × 200 mm, Sangon Biotech (Shanghai) Co., Ltd., “Sangon” for short) on water agar medium (WA), and incubated it at 28 °C for 24 h, and then selected the germinated single conidium under the stereoscopic microscope, transferred it to a new PDA medium for cultivation, and selected more than five single spores from each WA medium, selected a well-growing strain for standby. Scabs without conidia were isolated and purified using the tissue separation method [4]. The purified isolates were stored at 4 °C, PDA slants, and −80 °C, 20% glycerol for short-term and long-term storage. The information on isolates is shown in Table 7.

### 4.2. Morphological and Cultural Characterization

Morphology and cultural characterization followed the method of Diao et al. [15]. A 5 mm mycelial plug was taken from the edge of a vigorously growing colony and placed on a new 2% PDA plate. It was incubated for seven days under natural light at 28 °C, and then the colony’s diameter was measured, and color and texture were observed. After about one month, conidia pile, exudate, and sclerotia production were observed. For strains not prone to producing conidia, we used a culture with WA medium under the same conditions as PDA medium, with a cultivation time of 7–30 days. The shape, color, and size of setae, conidia, sporogenous cells, conidia appressoria, and mycelium appressoria were observed using the Olympus CX31 microscope (Olympus, Tokyo, Japan).

### 4.3. Pathogenicity Identification

The acupuncture inoculation method was used for inoculation identification. Healthy red ripe pepper fruits that had not been sprayed with fungicide were selected, disinfected with 75% alcohol, washed with sterile water, and dried. We used a sterilized toothpick to prick a wound at the part near the fruit stalk and the tip, with a diameter of about 1 mm, subjected to piercing the flesh. Each wound was inoculated with 1.0 × 10^5^ spores/mL conidia suspension 5 μL, using sterile water instead of spore suspension as a control treatment (abbreviated as CK). Each strain treated five pepper fruits, which were placed in a PP food preservation box covered with wet filter paper, and cultured at 25 °C for seven days. Then, the incidence of the fruit was observed and recorded. According to Koch’s formula, the pathogens on the diseased fruit were re-separated and purified, and whether the new isolate was the same as the inoculated pathogen was observed.

### 4.4. DNA Extraction, PCR Amplification, and DNA Sequencing

The aerial hyphae of the isolates cultured on a PDA plate for roughly 10 days were scraped, and DNA extracted using the plant genomic DNA kit (DP305) of Tiangen Biotech (Beijing) Co., Ltd., Beijing, China (from now on referred to as Tiangen). Firstly, the ITS sequence (ITS1/ITS4) [59] was amplified and sequenced, and tentative identification was established based on the NCBI comparison results and morphological assessment. The isolates that were identified as *Colletotrichum* spp. were further amplified for *ACT* (ACT-512F/ACT-783R) [60], *CHS-1* (CHS-79F/CHS-354R) [60], *GADPH* (GDFI/GDRI) [61], *TUB2* (T1/βt2b) [62,63], and *HIS3* (CYLH3F/CYLH3R) [64], and these PCR products were sent to Sangon for sequencing after detecting by electrophoresis on 1.2% agarose gel.

### 4.5. Phylogenetic Analysis

Using NCBI’s Blast tool to look for sequences with high homology and that belong to comparable pattern strains, the following sequences (Table 2, Table 3, Table 4 and Table 5) were compared using Cluster W to align. If necessary, Bioedit 7.2.6.1 was used for manual correction, and the corrected sequences were submitted to GenBank to receive accession numbers. The aligned sequences were concatenated by using SequenceMatrix-Windows-1.7.8 in the order ITS-ACT-CHS-1-GADPH-TUB2-HIS3. The concatenated sequences were translated using seaview4.0 format, and a phylogenetic tree was created using the Maximum Likelihood (ML) method in MEGA 6.06 [65]. In total, 1000 repeated bootstrap tests were conducted to establish branch support, which was not displayed when the support rate was less than 50%.

### 4.6. Genetic Diversity Analysis

In this study, there were a large number of isolates of *C. scovillei* and *C. fructicola*, and they had a wide distribution range, so genetic diversity analysis was conducted on the two species respectively. We took the spliced sequences used for phylogenetic tree construction as the analysis object and strains collected from different regions as different populations. The sequences’ base composition, variable sites (including gaps or missing sites in alignment, parsimony informative sites, and singleton variable sites), haplotype diversity, and fixation index (Fst) were analyzed using DNASP v5.0. Population dynamics were analyzed using Tajima’s test and Fu and Li’s test for neutrality testing. The correlation between genetic distance (GD) and geographical distance (GGD) was analyzed using GenAlEx 6.51b2. Additionally, a haplotype network diagram was constructed using Network 10.2.

## 5. Conclusions

This study found that the pathogen of Guizhou pepper anthracnose disease included 10 species: *C. scovillei*, *C. fructicola*, *C. karstii*, *C. truncatum*, *C. gloeosporioides*, *C. kahawae*, *C. boninense*, *C. nymphaeae*, *C. plurivorum*, and *C. nigrum*. *C. scovillei* and *C. fructicola* had a relatively large number of isolated strains, which might be the primary pathogenic fungi of pepper anthracnose in Guizhou. *C. nymphaeae* was isolated from Chinese chili peppers for the first time. Genetic diversity analysis has found that there might be population expansion in *C. fructicola*, which should be taken seriously in disease prevention and control.

## Figures and Tables

**Table 1:** Morphological characteristics of each *Colletotrichum* group.

Species	Colonies Appearance	Growth Rate (mm/day)	Conidiogenous Cells		Conidia			Conidial Appressoria			Mycelial Appressoria			Seta		
			Length (μm)	Width (μm)	Appearance	Length (μm)	Width (μm)	Appearance	Length (μm)	Width (μm)	Appearance	Length (μm)	Width (μm)	Appearance	Length (μm)	Width (μm)
Group 1 (*C. scovillei*)	white to orange, villous, produced dark gray sclerotia	6.9–11.6	7.55 ± 0.64 7.06–8.63	2.60 ± 0.25 2.35–2.94	hyaline, smooth-walled, aseptate, cylindrical or ovoid	9.27 ± 0.93 7.25–11.18	3.24 ± 0.29 2.75–0.92	nearly round, brown	5.11 ± 0.82 3.92–7.45	4.23 ± 0.53 3.14–5.29	cylindrical or irregular shape, occasionally in series	9.78 ± 3.55 3.92–019.22	4.92 ± 1.63 2.94–9.41	no		
Group 2 (*C. fructicola*)	white to dark gray, villous, reverse gray-green	5.4–5.6	15.54 ± 3.69 5.88–24.31	2.72 ± 0.50 1.96–3.92	colorless, cylindrical	12.63 ± 1.47 6.80–14.97	4.59 ± 0.37 4.08–5.44	brown to dark brown, ovoid or slightly irregular	7.32 ± 1.62 4.76–12.24	5.19 ± 0.79 4.08–8.16	cylindrical or round, some have deep or light cracks, brown or colorless, occasionally in series	10.39 ± 2.47 5.88–16.47	5.62 ± 1.41 3.14–8.24	light brown to dark brown, 2–4 cells, the base and top cells were light in color	60.55 ± 13.03 39.22–89.80	3.37 ± 0.59 2.35–4.51
Group 3 (*C. karstii*)	white, blanket-type	7.8–9.0	17.79 ± 5.0 29.80–27.45	3.27 ± 0.5 62.35–4.31	colorless, cylindrical	11.39 ± 1.19 9.22–15.29	5.05 ± 0.26 4.51–5.69	dark brown, nearly round or irregular	6.40 ± 0.54 5.10–7.65	5.53 ± 0.66 4.51–6.67	light brown to dark brown, cylindrical or irregular	8.94 ± 2.26 5.49–15.69 (21.96)	6.03 ± 1.49 4.31–10.98	no		
Group 4 (*C. truncatum*)	gray, villous, produced black sclerotia	8.3–12.5	not found		colorless, crescent-shaped with one rounded end and one acute end	16.50 ± 1.08 13.73–18.82	2.88 ± 0.19 2.35–3.14	nearly round, brown, sometimes in series	6.23 ± 0.74 4.71–7.84	4.38 ± 0.44 3.92–6.27	cylindrical or irregular, light brown to dark brown	7.34 ± 1.44 4.90–11.76	4.74 ± 0.70 3.92–7.45	2–4 cells, brown, the base and top cells were light in color	50.35 ± 11.1 834.90–67.06	3.57 ± 0.71 2.35–4.51
Group 5 (*C. gloeosporioides*)	gray, produced black sclerotia and orange conidial pile	9.11–12.1	not found		colorless, cylindrical or clavate	9.83 ± 2.36 6.80–12.93	3.24 ± 0.51 2.04–4.08	not found			nearly round or irregular, light brown to dark brown	8.37 ± 2.37 4.76–12.24	5.06 ± 0.97 3.40–6.80	no		
Group 6 (*C. kahawae*)	gray, blanket-type	7.2–8.1	9.78 ± 2.81 5.10–14.90 (21.57)	2.08 ± 0.52 1.18–3.14	colorless, cylindrical to clavate, both ends rounded or one end acute	10.20 ± 1.60 6.47–12.94	3.69 ± 0.52 2.55–4.71	brown, nearly round	5.65 ± 0.90 3.92–7.84	4.01 ± 0.34 3.53–4.71	light brown to dark brown, nearly round or irregular	9.46 ± 2.04 7.06–18.82	7.21 ± 1.59 4.71–11.37	no		
Group 7 (*C. boninense*)	milky white, produced brown to black sclerotia and a few conidia	7.9–11.0	12.11 ± 3.83 5.88–17.65	3.14 ± 0.99 1.96–5.29	hyaline, cylindrical, obtusely rounded at both ends	10.83 ± 1.50 7.84–13.73	5.57 ± 0.49 4.51–6.27	nearly round, brown	5.91 ± 1.30 3.92–9.80	4.14 ± 0.64 3.14–6.27	brown, cylindrical or irregular	11.16 ± 3.16 7.06–17.65	5.19 ± 0.90 3.73–6.67	no		
Group 8 (*C. nymphaeae*)	light gray, villous, produced dark gray sclerotia	7.0–8.8	13.04 ± 4.75 7.45–23.53	2.54 ± 0.55 1.96–3.73	colorless, cylindrical, obtusely rounded at both ends or tapered at one end	10.48 ± 1.02 7.65–11.96	3.88 ± 0.39 2.75–4.71	nearly round, brown	5.86 ± 0.97 3.53–7.45	3.95 ± 0.55 2.94–5.69	nearly round, brown	12.67 ± 3.18 8.24–19.61	3.48 ± 0.32 2.94–3.92	no		
Group 9 (*C. plurivorum*)	light gray to dark gray, blanket-type	4.7–8.4	11.76 ± 2.81 7.84–15.29	3.33 ± 0.37 2.75–3.73	colorless, cylindrical, obtusely rounded at both ends	14.52 ± 1.75 10.20–17.65	4.18 ± 0.33 3.53–5.10	brown or light brown, cylindrical or irregular	8.95 ± 1.41 5.49–12.16	4.86 ± 0.70 3.92–6.67	nearly cylindrical or irregular, brown or light brown	10.35 ± 1.86 7.45–13.33	6.11 ± 2.14 4.12–11.76	brown or light brown, 2–4 cells, and the base and top cells were light in color	70.78 ± 12.1 443.92–84.34	3.67 ± 0.52 3.14–4.90
Group 10 (*C. nigrum*)	light gray to dark gray, villous, produced black sclerotia	8.6–12.1	not found		hyaline, long cylindrical, obtusely rounded at both ends or tapered at one end	14.05 ± 1.43 9.41–17.45	3.66 ± 0.26 3.14–4.31	brown, nearly round	7.67 ± 1.33 5.10–11.76	4.55 ± 0.63 3.53–6.67	brown, cylindrical or irregular	10.54 ± 2.73 7.06–15.69	4.97 ± 0.88 3.14–6.27	brown or light brown, 2–4 cells, and the base and top cells were light in color	52.89 ± 9.69 39.22–73.73	3.96 ± 0.65 2.75–5.49

**Table 2:** The information on stains and isolates used for phylogenetic analysis of the *C. acutatum* species complex.

Species Name	Isolate	Host	Locality	GenBank Accessions					
				ITS	*ACT*	*CHS-1*	*GAPDH*	*TUB2*	*HIS3*
*C. acutatum*	CBS 112996 *	*Carica papaya*	Australia	JQ005776	JQ005839	JQ005797	JQ948677	JQ005860	JQ005818
	CBS 144.29	*Capsicum annuum*	Sri Lanka	JQ948401	JQ949722	JQ949062	JQ948732	JQ950052	JQ949392
*C. chrysanthemi*	CBS 126518	*Carthamus* sp.	Netherlands	JQ948271	JQ949592	JQ948932	JQ948601	JQ949922	JQ949262
	CBS 126519			JQ948272	JQ949593	JQ948933	JQ948602	JQ949923	JQ949263
*C. fioriniae*	CBS 128517 *	*Fiorinia* sp.	USA	JQ948292	JQ949613	JQ948953	JQ948622	JQ949943	JQ949283
*C. gloeosporioides*	IMI 356878 *	*Citrus sinensi*	Italy	JX010152	JX009531	JX009818	JX010056	JX010445	
*C. godetiae*	CBS 133.44 *	*Godetia* sp.	Denmark	JQ948402	JQ949723	JQ949063	JQ948733	JQ950053	JQ949393
*C. guajavae*	IMI 350839 *	*Psidium* sp.	India	JQ948270	JQ949591	JQ948931	JQ948600	JQ949921	JQ949261
*C. laticiphilum*	CBS 112989 *	*Hevea* sp.	India	JQ948289	JQ949610	JQ948950	JQ948619	JQ949940	JQ949280
*C. nymphaeae*	CBS 515.78 *	*Nymphaea* sp.	Netherlands	JQ948197	JQ949518	JQ948858	JQ948527	JQ949848	JQ949188
	**GL 17-90**	***Capsicum* sp.**	**China**	**OQ389348**	**OQ476130**	**OQ504687**	**OQ511698**	**OQ547942**	**OQ548022**
	**GL 17-92**	***Capsicum* sp.**	**China**	**OQ389349**	**OQ476129**	**OQ504688**	**OQ511699**	**OQ547943**	**OQ548023**
	**GL 21-90**	***Capsicum* sp.**	**China**	**OQ389350**		**OQ504689**	**OQ511700**	**OQ547944**	**OQ548024**
*C. salicis*	CBS 607.94 *	*Salix* sp.	Netherlands	JQ948460	JQ949781	JQ949121	JQ948791	JQ950111	JQ949451
*C. scovillei*	CBS 126529 *	*Capsicum* sp.	Indonesia	JQ948267	JQ949588	JQ948928	JQ948597	JQ949918	JQ949258
	CBS 126530	*Capsicum* sp.	Indonesia	JQ948268	JQ949589	JQ948929	JQ948598	JQ949919	JQ949259
	**GL_17-2**	***Capsicum* sp.**	**China**	**OQ389353**	**OQ476150**	**OQ504690**	**OQ511701**	**OQ547945**	**OQ548025**
	**GL_17-3**	***Capsicum* sp.**	**China**	**OQ389354**	**OQ476149**	**OQ504691**	**OQ511702**	**OQ547946**	**OQ548026**
	**GL_17-12**	***Capsicum* sp.**	**China**	**OQ389355**	**OQ476148**	**OQ504692**		**OQ547947**	**OQ548027**
	**GL_17-18**	***Capsicum* sp.**	**China**	**OQ389356**	**OQ476147**	**OQ504693**	**OQ511703**	**OQ547948**	**OQ548028**
	**GL_17-24**	***Capsicum* sp.**	**China**	**OQ389357**		**OQ504694**	**OQ511704**		**OQ548029**
	**GL_17-38**	***Capsicum* sp.**	**China**	**OQ389358**	**OQ476146**	**OQ504695**	**OQ511705**	**OQ547949**	**OQ548030**
	**GL_17-66**	***Capsicum* sp.**	**China**	**OQ389359**	**OQ476145**	**OQ504696**	**OQ511706**	**OQ547958**	**OQ548031**
	**GL_17-73**	***Capsicum* sp.**	**China**	**OQ389360**	**OQ476144**	**OQ504697**	**OQ511707**	**OQ547950**	**OQ548032**
	**GL_17-86**	***Capsicum* sp.**	**China**	**OQ389361**	**OQ476143**	**OQ504698**	**OQ511708**	**OQ547959**	**OQ548033**
	**GL_17-93**	***Capsicum* sp.**	**China**	**OQ389362**	**OQ476142**	**OQ504699**		**OQ547951**	**OQ548034**
	**GL_17-101**	***Capsicum* sp.**	**China**	**OQ389351**	**OQ476141**	**OQ504700**	**OQ511709**	**OQ547952**	**OQ548035**
	**GL_17-108**	***Capsicum* sp.**	**China**	**OQ389352**	**OQ476140**	**OQ504701**	**OQ511710**	**OQ547960**	**OQ548036**
	**GL_17-114**	***Capsicum* sp.**	**China**	**OQ389363**	**OQ476139**	**OQ504702**	**OQ511711**	**OQ547961**	**OQ548037**
	**GL_17-115**	***Capsicum* sp.**	**China**	**OQ389364**	**OQ476138**	**OQ504703**		**OQ547962**	**OQ548038**
	**GL_17-130**	***Capsicum* sp.**	**China**	**OQ389365**	**OQ476137**	**OQ504704**	**OQ511712**	**OQ547963**	**OQ548039**
	**GL_17-157**	***Capsicum* sp.**	**China**	**OQ389366**	**OQ476136**	**OQ504705**		**OQ547964**	**OQ548040**
	**GL_17-169**	***Capsicum* sp.**	**China**	**OQ389367**	**OQ476135**	**OQ504706**		**OQ547965**	**OQ548041**
	**GL_21-35**	***Capsicum* sp.**	**China**	**OQ389368**		**OQ504707**	**OQ511713**	**OQ547953**	**OQ548042**
	**GL_21-127**	***Capsicum* sp.**	**China**	**OQ389369**	**OQ476131**	**OQ504708**	**OQ511714**	**OQ547954**	**OQ548043**
	**GL_21-144**	***Capsicum* sp.**	**China**	**OQ389370**	**OQ476132**	**OQ504709**	**OQ511715**	**OQ547955**	**OQ548044**
	**GL_21-186**	***Capsicum* sp.**	**China**	**OQ389371**	**OQ476133**	**OQ504710**	**OQ511716**	**OQ547956**	**OQ548045**
	**GL_21-206**	***Capsicum* sp.**	**China**	**OQ389372**	**OQ476134**	**OQ504711**	**OQ511717**	**OQ547957**	**OQ548046**
*C. simmondsii*	CBS 122122 *	*Carica* sp.	Australia	JQ948276	JQ949597	JQ948937	JQ948606	JQ949927	JQ949267

Notes: The strains with “*” were ex-type or ex-epitype cultures; isolates studied in this paper are in bold font.

**Table 3:** The information on stains and isolates used for phylogenetic analysis of the *C. gloeosporioides* species complex.

Species Name	Isolate	Host	Locality	GenBank Accessions					
				ITS	*ACT*	*CHS-1*	*GAPDH*	*TUB2*	*HIS3*
*C. aenigma*	ICMP 18608 *	*Persea americana*	Israel	JX010244	JX009443	JX009789	JX010044	JX010389	
*C. alienum*	ICMP 12071 *	*Malus domestica*	New Zealand	JX010251	JX009572	JX009882	JX010028	JX010411	
*C. aotearoa*	ICMP 18537 *	*Coprosma* sp.	New Zealand	JX010205	JX009564	JX009853	JX010005	JX010420	
*C. asianum*	ICMP 18580, CBS 130418 *	*Coffea arabica*	Thailand	FJ972612	JX009584	JX009867	JX010053	JX010406	KY856305
*C. camelliae*	CGMCC 3.14925, LC 1364 *	*Camillia sinensis*	China	KJ955081	KJ954363		KJ954782	KJ955230	MZ673847
*C. clidemiae*	ICMP 18658 *	*Clidemia hirta*	USA, Hawaii	JX010265	JX009537	JX009877	JX009989	JX010439	
*C. conoides*	CAUG 17 *	*Capsicum* sp.	China	KP890168	KP890144	KP890156	KP890162	KP890174	
*C. fructicola*	CBS 125397 *	*Tetragastris panamensis*	Panama	JX010173	JX009581	JX009874	JX010032	JX010409	KY856315
	ICMP 18581, CBS130416 *	*Coffea arabica*	Thailand	JX010165	FJ907426	JX009866	JX010033	JX010405	
	**GL 17-29**	***Capsicum* sp.**	**China**	**OQ389311**	**OQ476097**	**OQ504650**	**OQ511663**	**OQ547912**	**OQ547987**
	**GL 17-45**	***Capsicum* sp.**	**China**	**OQ389312**	**OQ476101**	**OQ504651**	**OQ511664**	**OQ547913**	
	**GL 17-67**	***Capsicum* sp.**	**China**	**OQ389313**	**OQ476102**	**OQ504652**		**OQ547914**	**OQ547988**
	**GL 17-153**	***Capsicum* sp.**	**China**	**OQ389314**	**OQ476096**	**OQ504653**	**OQ511673**		**OQ547989**
	**GL 21-24**	***Capsicum* sp.**	**China**	**OQ389315**	**OQ476108**	**OQ504655**	**OQ511665**	**OQ547915**	**OQ547990**
	**GL 21-43**	***Capsicum* sp.**	**China**	**OQ389316**	**OQ476106**	**OQ504656**	**OQ511666**	**OQ547916**	**OQ547991**
	**GL 21-56-1**	***Capsicum* sp.**	**China**	**OQ389317**		**OQ504657**	**OQ511667**	**OQ547917**	**OQ547992**
	**GL 21-75**	***Capsicum* sp.**	**China**	**OQ389318**	**OQ476109**	**OQ504658**	**OQ511668**	**OQ547918**	**OQ547993**
	**GL 21-101**	***Capsicum* sp.**	**China**	**OQ389319**	**OQ476098**	**OQ504654**	**OQ511669**		**OQ547994**
	**GL 21-122-1**	***Capsicum* sp.**	**China**	**OQ389320**	**OQ476103**	**OQ504659**	**OQ511670**	**OQ547919**	**OQ547995**
	**GL 21-130-4**	***Capsicum* sp.**	**China**	**OQ389321**	**OQ476104**	**OQ504660**	**OQ511671**	**OQ547920**	**OQ547996**
	**GL 21-145**	***Capsicum* sp.**	**China**	**OQ389322**	**OQ476105**	**OQ504661**	**OQ511672**	**OQ547921**	**OQ547997**
	**GL 21-177**	***Capsicum* sp.**	**China**	**OQ389323**	**OQ476100**	**OQ504662**	**OQ511674**	**OQ547922**	**OQ547998**
	**GL 21-221**	***Capsicum* sp.**	**China**	**OQ389324**	**OQ476107**	**OQ504663**	**OQ511675**	**OQ547923**	**OQ547999**
	**GL 21-225**	***Capsicum* sp.**	**China**	**OQ389325**	**OQ476099**	**OQ504664**	**OQ511676**	**OQ547924**	**OQ548000**
*C. gloeosporioides*	CAUG 19	*Capsicum* sp.	China	KP145432	KP145320	KP145376	KP145404	KP145460	
	IMI 356878 *	*Citrus sinensis*	Italy	NR160754	JX009531	JX009818	JX010056	JX010445	
	**GL 17-72**	***Capsicum* sp.**	**China**	**OQ389328**	**OQ476110**	**OQ504667**	**OQ511677**	**OQ547925**	**OQ548001**
	**GL 17-102**	***Capsicum* sp.**	**China**	**OQ389327**	**OQ476111**	**OQ504665**	**OQ511678**	**OQ547926**	**OQ548002**
	**GL 17-107**	***Capsicum* sp.**	**China**	**OQ389326**	**OQ476112**	**OQ504666**	**OQ511679**	**OQ547927**	**OQ548003**
	**GL 21-22-1**	***Capsicum* sp.**	**China**	**OQ389329**		**OQ504668**		**OQ547928**	**OQ548004**
*C. grevilleae*	CBS 132879 *	*Grevillea* sp.	Italy	KC297078	KC296941	KC296987	KC297010	KC297102	
*C. grossum*	CAUG 7 *	*Capsicum* sp.	China	KP890165	KP890141	KP890153	KP890159	KP890171	KC297056
*C. kahawae*	subsp. cigarro CBS 237.49	*Hypericum perforatum*	Germany	JX010238	JX009450	JX009840	JX010042	JX010432	
	subsp. kahawae ICMP 17816	*Coffea arabica*	Kenya	JX010231	JX009452	JX009813	JX010012	JX010444	
	**GL 17-100**	***Capsicum* sp.**	**China**	**OQ389330**	**OQ476113**	**OQ504669**	**OQ511680**	**OQ547929**	**OQ548005**
	**GL 21-95**	***Capsicum* sp.**	**China**	**OQ389331**	**OQ476114**	**OQ504670**	**OQ511681**	**OQ547930**	**OQ548006**
	**GL 21-152**	***Capsicum* sp.**	**China**	**OQ389332**	**OQ476115**	**OQ504671**	**OQ511682**		**OQ548007**
	**GL 21-169**	***Capsicum* sp.**	**China**	**OQ389333**	**OQ476116**	**OQ504672**	**OQ511683**		**OQ548008**
	**GL 21-196**	***Capsicum* sp.**	**China**	**OQ389334**	**OQ476117**	**OQ504673**	**OQ511684**	**OQ547931**	**OQ548009**
	**GL 21-218**	***Capsicum* sp.**	**China**	**OQ389335**	**OQ476118**	**OQ504674**	**OQ511685**	**OQ547932**	
	**GL 21-219**	***Capsicum* sp.**	**China**	**OQ389336**	**OQ476119**	**OQ504675**	**OQ511686**	**OQ547933**	**OQ548010**
	**GL 21-223-2**	***Capsicum* sp.**	**China**	**OQ389337**	**OQ476120**	**OQ504676**	**OQ511687**	**OQ547934**	**OQ548011**
*C. queenslandicum*	ICMP 1778 *	*Carica papaya*	Australia	JX010276	JX009447	JX009899	JX009934	JX010414	
*C. siamense*	CBS 125378	*Hymenocallis americana*	China	JX010278	JX009441		JX010019	JX010410	
*C. ti*	ICMP 4832 *	*Cordyline* sp.	New Zealand	JX010269	JX009520	JX009898	JX009952	JX010442	
*C. tropicale*	CBS 124949 *	*Theobroma cacao*	Panama	JX010264	JX009489	JX009870	JX010007	JX010407	KY856395
*C. liaoningense*	CAUOS 2 *	*Capsicum* sp.	China	KP890104	KP890097	KP890127	KP890135	KP890111	

Notes: The strains with “*” were ex-type cultures; isolates studied in this paper are in bold font.

**Table 4:** The information on stains and isolates used for phylogenetic analysis of the *C. boninense* species complex.

Species Name	Isolate	Host	Locality	GenBank Accessions					
				ITS	*ACT*	*CHS-1*	*GAPDH*	*TUB2*	*HIS3*
*C. annellatum*	CBS 129826 *	*Hevea brasiliensis*	Colombia	JQ005222	JQ005570	JQ005396	JQ005309	JQ005656	JQ005483
*C. beeveri*	CBS 128527 *	*Brachyglottis repanda*	New Zealand	JQ005171	JQ005519	JQ005345	JQ005258	JQ005605	JQ005432
*C. boninense*	CBS 123755 *	*Crinum asiaticum* var. *sinicum*	Japan	JQ005153	JQ005501	JQ005327	JQ005240	JQ005588	JQ005414
	MAFF 305972, ICMP 17904 *	*Crinum asiaticum* var. *sinicum*	Japan	JX010292	JX009583		JX009905		
	**GL 17-121**	***Capsicum* sp.**	**China**	**OQ389303**	**OQ476092**		**OQ511659**	**OQ547908**	**OQ547979**
	**GL 21-102-1**	***Capsicum* sp.**	**China**	**OQ389304**	**OQ476088**	**OQ504646**	**OQ511655**		**OQ547980**
	**GL 21-166**	***Capsicum* sp.**	**China**	**OQ389305**	**OQ476089**		**OQ511656**		**OQ547981**
	**GL 21-193**	***Capsicum* sp.**	**China**	**OQ389306**	**OQ476090**	**OQ504644**	**OQ511657**		**OQ547982**
	**GL 21-217-2**	***Capsicum* sp.**	**China**	**OQ389307**	**OQ476091**	**OQ504645**	**OQ511658**		**OQ547983**
*C. brasiliense*	CBS 128501 *	*Passiflora edulis* f. *flavicarpa*	Brazil	JQ005235	JQ005583	JQ005409	JQ005322	JQ005669	JQ005414
*C. brassicicola*	CBS 101059 *	*Brassica oleracea* var. *gemmifera*	New Zealand	JQ005172	JQ005520	JQ005346	JQ005259	JQ005606	JQ005496
*C. camelliae-japonicae*	CGMCC 3.18118 *	*Camellia japonica*	Japan	KX853165	KX893576		KX893584	KX893580	JQ005433
*C. catinaense*	CBS 142417 *	*Citrus reticulata*	Italy	KY856400	KY855971	KY856136	KY856224	KY856482	
*C. citricola*	CBS 134228 *	*Citrus unshiu*	China	KC293576	KC293616	KC293792	KC293736	KC293656	KY856311
*C. colombiense*	CBS 129818 *	*Passiflora edulis*	Colombia	JQ005174	JQ005522	JQ005348	JQ005261	JQ005608	KY856311
*C. condaoense*	CBS 134299 *	*Ipomoea pescaprae*	Vietnam	MH229914		MH229926	MH229920	MH229923	JQ005435
*C. cymbidiicola*	IMI 347923 *	*Cymbidium* sp.	Australia	JQ005166	JQ005514	JQ005340	JQ005253	JQ005600	JQ005499
*C. feijoicola*	CBS 144633 *	*Acca sellowiana*	Portugal	MK876413	MK876466		MK876475	MK876507	
*C. hippeastri*	CBS 125376 *	*Hippeastrum vittatum*	China	JQ005231	JQ005579	JQ005405	JQ005318	JQ005665	
*C. karstii*	CORCG 6	*Vanda* sp.	China	HM585409	HM581995	HM582023	HM585391	HM585428	JQ005492
	**GL_17-71**	***Capsicum* sp.**	**China**	**OQ389338**		**OQ504677**	**OQ511688**		**OQ548012**
	**GL_17-159**	***Capsicum* sp.**	**China**	**OQ389339**		**OQ504678**	**OQ511689**		**OQ548013**
	**GL_21-36-2**	***Capsicum* sp.**	**China**	**OQ389340**	**OQ476128**	**OQ504679**	**OQ511690**	**OQ547935**	**OQ548014**
	**GL_21-44**	***Capsicum* sp.**	**China**	**OQ389341**	**OQ476121**	**OQ504680**	**OQ511691**	**OQ547936**	**OQ548015**
	**GL_21-116-3**	***Capsicum* sp.**	**China**	**OQ389342**	**OQ476127**	**OQ504681**	**OQ511692**	**OQ547937**	**OQ548016**
	**GL_21-120-1**	***Capsicum* sp.**	**China**	**OQ389343**	**OQ476122**	**OQ504682**	**OQ511693**		**OQ548017**
	**GL_21-199**	***Capsicum* sp.**	**China**	**OQ389344**	**OQ476123**	**OQ504683**	**OQ511694**	**OQ547938**	**OQ548018**
	**GL_21-202-2**	***Capsicum* sp.**	**China**	**OQ389345**	**OQ476124**	**OQ504684**	**OQ511695**	**OQ547939**	**OQ548019**
	**GL_21-211-4**	***Capsicum* sp.**	**China**	**OQ389346**	**OQ476125**	**OQ504685**	**OQ511696**	**OQ547940**	**OQ548020**
	**GL_21-216**	***Capsicum* sp.**	**China**	**OQ389347**	**OQ476126**	**OQ504686**	**OQ511697**	**OQ547941**	**OQ548021**
*C. limonicola*	CBS 142410 *	*Citrus limon*	Malta	KY856472	KY856045	KY856213	KY856296	KY856554	
*C. novae-zelandiae*	CBS 128505 *	*Capsicum annuum*	New Zealand	JQ005228	JQ005576	JQ005402	JQ005315	JQ005662	KY856388
*C. parsonsiae*	CBS 128525 *	*Parsonsia capsularis*	New Zealand	JQ005233	JQ005581	JQ005407	JQ005320	JQ005667	JQ005430
*C. petchii*	CBS 378.94 *	*Dracaena fragrans*	Italy	JQ005223	JQ005571	JQ005397	JQ005310	JQ005657	JQ005494
*C. phyllanthi*	CBS 175.67 *	*Phyllanthus acidus*	India	JQ005221	JQ005569	JQ005395	JQ005308	JQ005655	JQ005484
*C. gloeosporioides*	IMI 356878 *	*Citrus sinensi*	Italy	JX010152	JX009531	JX009818	JX010056	JX010445	

Notes: The strains with “*” were ex-type cultures; isolates studied in this paper are in bold font.

**Table 5:** The information on stains and isolates used for phylogenetic analysis of the *C. truncatum* species complex and other species.

Species Name	Isolate	Host	Locality	GenBank Accessions					
				ITS	*ACT*	*CHS-1*	*GAPDH*	*TUB2*	*HIS3*
*C. acidae*	MFLUCC 17-2659 *	*Phyllanthus acidus*	Thailand	MG996505	MH003697	MH003694	MH003691	MH003700	
*C. cattleyicola*	CBS 17049 *	*Cattleya* sp.	Belgium	MG600758	MG600963	MG600866	MG600819	MG601025	MG600905
*C. chlorophyti*	IMI 103806 *	*Chlorophytum* sp.	India	GU227894	GU227992	GU228384	GU228286	GU228188	GU228090
*C. cliviicola*	CBS 125375	*Clivia miniata*	China	MG600733	MG600939	MG600850	MG600795	MG601000	
*C. citrus-medicae*	GUCC 1554	*Citrus medica*	China	MN959910	MT006325	MT006328	MT006331	OQ547911	MT006334
*C. coccodes*	CBS 369.75 *	*Solanum tuberosum*	Netherlands	HM171679	HM171667	JX546681	HM171673	JX546873	JX546779
*C. curcumae*	IMI 288937 *	*Curcuma longa*	India	GU227893	GU227991	GU228383	GU228285	GU228187	GU228089
*C. fusiforme*	MFLU 13-0291 *	*Homo sapiens*	Thailand	KT290266	KT290251	KT290253	KT290255	KT290256	
*C. musicola*	CBS 132885	*Musa* sp.	Mexico	MG600736	MG600942	MG600853	MG600798	MG601003	MG600895
*C. nigrum*	CBS 169.49 *	*Capsicum* sp.	Argentina	JX546838	JX546646	JX546693	JX546742	JX546885	JX546791
	**GL 21-121**	***Capsicum* sp.**	**China**	**OQ389310**	**OQ476095**	**OQ504649**	**OQ511662**	**OQ547911**	**OQ547986**
*C. orchidearum*	CBS 135131	*Dendrobium nobile*	Netherlands	MG600738	MG600944	MG600855	MG600800	MG601005	MG600897
*C. phaseolorum*	CBS 158.36	*Vigna sinensis*	Japan	GU227897	GU227995	GU228387	GU228289	GU228191	GU228093
*C. plurivorum*	CBS 125474 *	*Coffea* sp.	Vietnam	MG600718	MG600925	MG600841	MG600781	MG600985	MG600887
	**GL 21-67**	***Capsicum* sp.**	**China**	**OQ389308**	**OQ476093**	**OQ504647**	**OQ511660**	**OQ547909**	**OQ547984**
	**GL 21-183**	***Capsicum* sp.**	**China**	**OQ389309**	**OQ476094**	**OQ504648**	**OQ511661**	**OQ547910**	**OQ547985**
*C. pseudoacutatum*	CBS 436.77 *	*Pinus radiata*	Chile	JQ948480	JQ949801	JQ949141	JQ948811	JQ950131	JQ949471
*C. piperis*	IMI 71397 *	*Piper nigrum*	Malaysia	MG600760	MG600964	MG600867	MG600820	MG601027	MG600906
*C. pyrifoliae*	CGMCC 3.18902, PAFQ22	*Pyrus pyrifolia*	China	MG748078	MG747768	MG747914	MG747996	MG748158	
*C. rusci*	CBS 119206	*Ruscus*	Italy	GU227818	GU227916	GU228308	GU228210	GU228112	GU2280141
*C. sojae*	ATCC 62257	*Glycine max*	USA	MG600749	MG600954	MG600860	MG600810	MG601016	KC110803
*C. sydowii*	CBS135819 *	Sambucus	China: Taiwan	KY263783	KY263791	KY263787	KY263785	KY263793	KY263789
*C. trichellum*	CBS 217.64 *	*Hedera helix*	UK	GU227812	GU227910	GU228302	GU228204	GU228106	
*C. truncatum*	CBS 151.35 *	*Phaseolus lunatus*	USA	GU227862	GU227960	GU228352	GU228254	GU228156	GU228058
	**GL 17-59**	***Capsicum* sp.**	**China**	**OQ389373**	**OQ476151**	**OQ504712**		**OQ547971**	**OQ548047**
	**GL 17-60**	***Capsicum* sp.**	**China**	**OQ389374**	**OQ476152**	**OQ504713**	**OQ511718**	**OQ547966**	
	**GL 17-87**	***Capsicum* sp.**	**China**	**OQ389375**	**OQ476153**	**OQ504714**	**OQ511719**	**OQ547972**	**OQ548048**
	**GL 17-154**	***Capsicum* sp.**	**China**	**OQ389376**	**OQ476154**	**OQ504715**		**OQ547967**	**OQ548049**
	**GL 17-162**	***Capsicum* sp.**	**China**	**OQ389377**	**OQ476155**	**OQ504716**	**OQ511720**	**OQ547968**	**OQ548050**
	**GL 17-163**	***Capsicum* sp.**	**China**	**OQ389378**	**OQ476156**	**OQ504717**	**OQ511721**	**OQ547969**	**OQ548051**
	**GL 17-171**	***Capsicum* sp.**	**China**	**OQ389379**	**OQ476157**	**OQ504718**		**OQ547970**	**OQ548052**
	**GL 21-30-1**	***Capsicum* sp.**	**China**	**OQ389380**	**OQ476158**	**OQ504719**	**OQ511728**	**OQ547973**	**OQ548053**
	**GL 21-31-1**	***Capsicum* sp.**	**China**	**OQ389381**	**OQ476159**	**OQ504720**	**OQ511722**		**OQ548054**
	**GL 21-32-2**	***Capsicum* sp.**	**China**	**OQ389382**	**OQ476160**	**OQ504721**	**OQ511723**		**OQ548055**
	**GL 21-46**	***Capsicum* sp.**	**China**	**OQ389383**	**OQ476161**	**OQ504722**	**OQ511724**	**OQ547974**	**OQ548056**
	**GL 21-200**	***Capsicum* sp.**	**China**	**OQ389384**	**OQ476162**	**OQ504723**	**OQ511725**	**OQ547975**	**OQ548057**
	**GL 21-203**	***Capsicum* sp.**	**China**	**OQ389385**	**OQ476163**	**OQ504724**	**OQ511726**	**OQ547976**	**OQ548058**
	**GL 21-208**	***Capsicum* sp.**	**China**	**OQ389386**	**OQ476164**	**OQ504725**	**OQ511729**	**OQ547977**	**OQ548059**
	**GL 21-212**	***Capsicum* sp.**	**China**	**OQ389387**	**OQ476165**	**OQ504726**	**OQ511727**	**OQ547978**	**OQ548060**
*C. vittalense*	CBS 181.82	*Theobroma cacao*	India	MG600734	MG600940	MG600851	MG600796	MG601001	MG600893
*Monilochaetes infuscans*	CBS 869.96 *	*Ipormoea batatas*	South Africa	JQ005780	JQ005843	JQ005801	JX546612	JQ005864	JQ005822

Notes: The strains with “*” were ex-type cultures; isolates studied in this paper are in bold font.

**Table 6:** Fst of *C. fructicola* between different populations.

Population	BJ	GY	LPS	QDN	QN	ZY
GY	0.3143					
LPS	0.7500	0.4267				
QDN	0.6092	0.3887	0.0769			
QN	0.8091	0.1449	0.8633	0.8441		
ZY	0.0625	0.0874	0.3974	0.2967	0.6363	
TR	0.6667	0.4034	0.0000	0.0634	0.8513	0.3366

**Table 7:** The list of all *Colletotrichum* spp. collected from pepper in Guizhou based on preliminary identification.

Species	Location	Host Tissue	Number of Isolates	Longitude	Latitude
*C. scovillei*	Huaxi, Guiyang	Fruit	1	106.66	26.50
	Honghuagang, Zunyi	Fruit	30	106.89	27.64
	Bozhou, Zunyi	Fruit	16	106.83	27.54
	Suiyang, Zunyi	Fruit	5	107.19	27.95
	Hezhang, Bijie	Fruit	16	104.83	27.12
	Dafang, Bijie	Fruit	3	105.61	27.14
	Puding, Anshun	Fruit	4	105.74	26.30
	Sandu, Qiannan	Fruit	6	107.90	25.62
	Pingtang, Qiannan	Fruit	7	107.24	25.83
	Fuquan, Qiannan	Fruit	8	107.63	27.63
	Changshun, Qiannan	Fruit	7	106.45	26.02
	Huangping, Qiandongnan	Fruit	7	107.92	26.91
	Liuzhi, Liupanshui	Fruit	40	108.58	28.04
*C. fructicola*	Huaxi, Guiyang	Fruit	4	106.66	26.50
	Xiuwen, Guiyang	Fruit	3	106.59	26.84
	Honghuagang, Zunyi	Fruit	1	106.89	27.64
	Bozhou, Zunyi	Fruit	11	106.83	27.54
	Suiyang, Zunyi	Fruit	1	107.19	27.95
	Qixingguan, Bijie	Fruit	1	105.30	27.30
	Dafang, Bijie	Fruit	2	105.61	27.14
	Puding, Anshun	Fruit	1	105.74	26.30
	Yinjiang, Tongren	Fruit	13	108.41	27.99
	Sandu, Qiannan	Fruit	1	107.90	25.62
	Pingtang, Qiannan	Fruit	13	107.24	25.83
	Huangping, Qiandongnan	Fruit	17	107.92	26.91
	Liuzhi, Liupanshui	Fruit	6	108.58	28.04
*C. karstii*	Xiuwen, Guiyang	Fruit	1	106.59	26.84
	Honghuagang, Zunyi	Fruit	1	106.89	27.64
	Bozhou, Zunyi	Fruit	4	106.83	27.54
	Suiyang, Zunyi	Fruit	6	107.19	27.95
	Dafang, Bijie	Fruit	6	105.61	27.14
	Yinjiang, Tongren	Fruit	1	108.41	27.99
*C. truncatum*	Honghuagang, Zunyi	Fruit	3	106.89	27.64
	Bozhou, Zunyi	Fruit	1	106.83	27.54
	Suiyang, Zunyi	Fruit, leaf	3	107.19	27.95
	Dafang, Bijie	Fruit	6	105.61	27.14
	Yinjiang, Tongren	Fruit	1	108.41	27.99
	Huangping, Qiandongnan	Fruit	1	107.92	26.91
	Liuzhi, Liupanshui	Fruit	2	108.58	28.04
*C. gloeosporioides*	Huaxi, Guiyang	Fruit	7	106.66	26.50
	Bozhou, Zunyi	Fruit	5	106.89	27.64
*C. kahawae*	Suiyang, Zunyi	Fruit	3	106.83	27.54
	Dafang, Bijie	Fruit	1	107.19	27.95
	Ziyun, Anshun	Fruit	1	106.08	25.75
	Yinjiang, Tongren	Fruit	3	108.41	27.99
	Sandu, Qiannan	Fruit	2	107.90	25.62
*C. boninense*	Honghuagang, Zunyi	Fruit	2	106.89	27.64
	Qixingguan, Bijie	Fruit	3	105.30	27.30
	Yinjiang, Tongren	Fruit	1	108.41	27.99
	Sandu, Qiannan	Fruit	1	107.90	25.62
	Liuzhi, Liupanshui	Fruit	1	108.58	28.04
*C. nymphaeae*	Ziyun, Anshun	Fruit	1	106.08	25.75
	Huangping, Qiandongnan	Fruit	2	107.92	26.91
*C. plurivorum*	Pingtang, Qiannan	Fruit	1	107.24	25.83
	Huangping, Qiandongnan	Fruit	1	107.92	26.91
*C. nigrum*	Xiuwen, Guiyang	Fruit	1	106.59	26.84
Total			296		

## Data Availability

Publicly available datasets were analyzed in this study. These data can be found here: https://www.ncbi.nlm.nih.gov/ (accessed on 1 December 2022).
